# Cross-protection against homo and heterologous influenza viruses via intranasal administration of an HA chimeric multiepitope nanoparticle vaccine

**DOI:** 10.1186/s12951-025-03122-6

**Published:** 2025-02-04

**Authors:** Yongqiang Zhao, Jia Liu, Chun Peng, Shuangshuang Guo, Bo Wang, Longping Chen, Yating Wang, Haiwen Tang, Liming Liu, Qi Pan, Shiren Li, Jingyu Wang, Dongni Yang, Enqi Du

**Affiliations:** 1 College of Veterinary Medicine, Northwest A&F University, Yangling, Shaanxi 712100 China; 2 Yangling Carey Biotechnology Co., Ltd., Yangling, Shaanxi 712100 China; 3 Chengdu NanoVAX Biotechnology Co., Ltd., Chengdu, Sichuan 610219 China; 4 Nanjing JSIAMA Biopharmaceuticals Ltd., Nanjing, Jiangsu 210000 China

**Keywords:** Influenza virus, Nanoparticle vaccine, Universal influenza vaccine, Cross-protection

## Abstract

**Background:**

Influenza A viruses (IAVs) cause seasonal influenza epidemics and pose significant threats to public health. However, seasonal influenza vaccines often elicit strain-specific immune responses and confer little protection against mismatched strains. There is an urgent need to develop universal influenza vaccines against emerging and potentially re-emerging influenza virus infections. Multiepitope vaccines combining multiple conserved epitopes can induce more robust and broader immune responses and provide a potential solution.

**Results:**

Here, we demonstrated that an HA chimeric multiepitope nanoparticle vaccine, delivered intranasally conferred broad protection against challenges with various influenza viruses in mice. The nanoparticle vaccine co-expresses the ectodomain of haemagglutinin (H), three repeated highly conserved ectodomains of matrix protein 2 (M), and the M-cell-targeting ligand Co4B (C) in a baculovirus-insect cell system. These elements (C, H and M) were presented on the surface of self-assembling ferritin (f) in tandem to generate a nanoparticle denoted as CHM-f. Intranasal vaccination with CHM-f nanoparticles elicited robust humoral and cellular immune responses, conferring complete protection against a variety of IAVs, including the A/PR8/34 H1N1 strain, the swine flu H3N2 strain, the avian flu H5N8 strain, and H9N2. When CHM-f nanoparticles adjuvanted with CpG IAMA-002, the weight loss protective effect, cellular immune responses and mucosal IgA responses were significantly augmented. Compared with controls, mice immunized with CHM-f nanoparticles with or without CpG IAMA-002 showed significant reductions in weight loss, lung viral titres and pathological changes.

**Conclusions:**

These results suggest that CHM-f nanoparticle with or without CpG IAMA-002 is a promising candidate as a universal influenza vaccine.

**Graphical Abstract:**

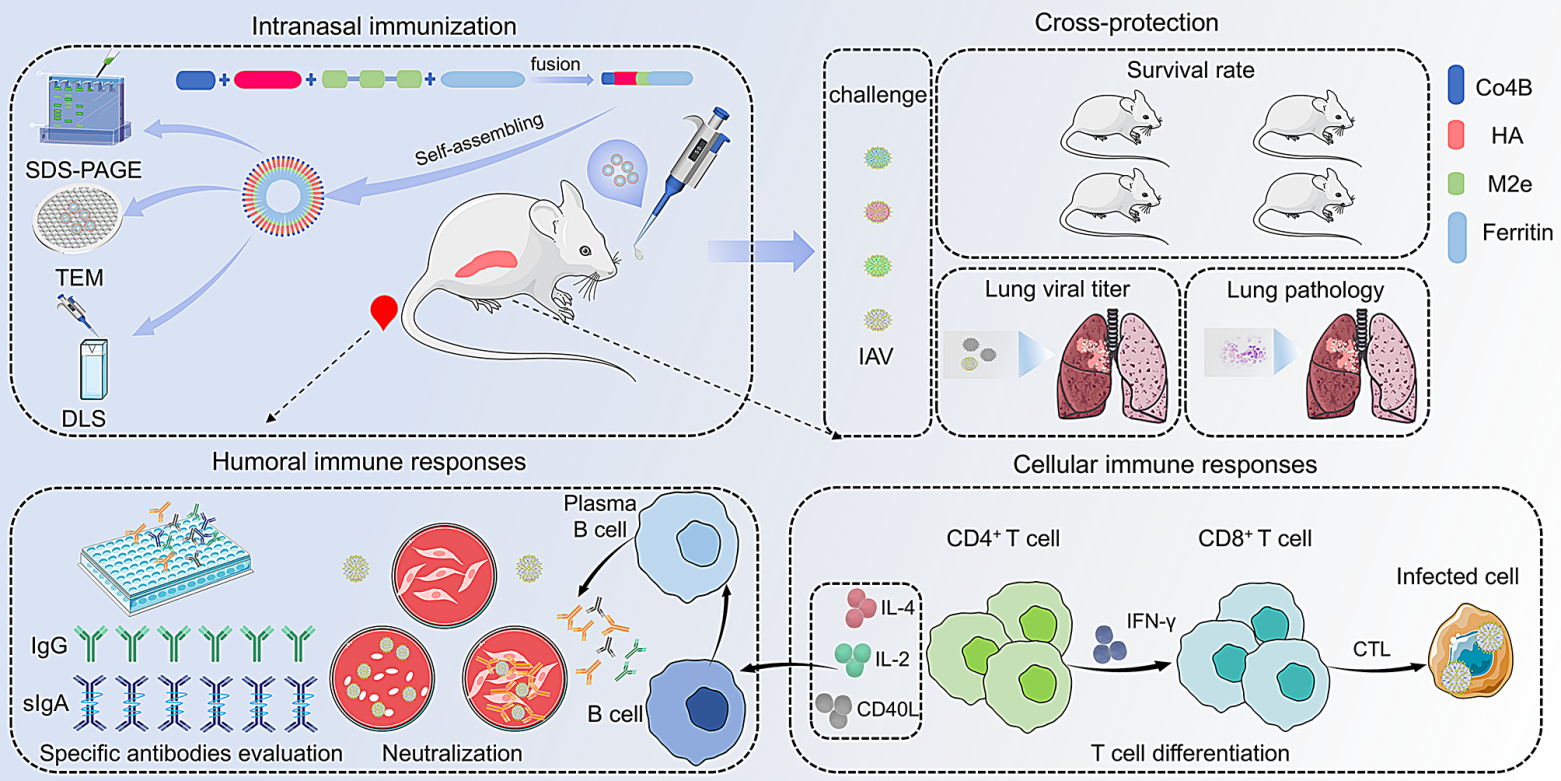

**Supplementary Information:**

The online version contains supplementary material available at 10.1186/s12951-025-03122-6.

## Background

Influenza is a widespread acute respiratory infection that poses a significant burden on public health. Seasonal influenza causes approximately 3–5 million cases of critical illness worldwide, with 290–650 thousand deaths annually [1]. Influenza vaccination is at present the most cost-effective strategy to control influenza. However, current seasonal influenza vaccines provide only 10-60% partial protection against matched strains, with little or no protection against mismatched strains [2]. Frequent antigenic drift and shifts in influenza viruses severely compromise the protective efficacy of the original vaccine. Therefore, there is an urgent need to develop a universal vaccine that is effective against both emerging and potentially re-emerging influenza viruses.

Influenza viruses invade the body through mucosal surfaces, which constitute the first line of defense against pathogen invasion. The mucosal immune system is not only an important part of the whole immune network but is also an independent immune system with a unique structure and function. Nasal vaccines can induce not only a mucosal but also a systemic immune response, and can play an immunoprotective role in the early stage of pathogen invasion, effectively overcoming the limitations of existing vaccines [3]. Despite the many advantages of mucosal immunization, a limited number of influenza nasal vaccines are licensed. And, they use live attenuated influenza viruses (LAIVs) [4, 5] which pose a potential risk of genetic reassortment and reversion of virulence in high-risk populations, such as infants under 2 years of age and elderly individuals over 60 years of age. Compared with LAIVs, subunit vaccines use viral antigens obtained through in vitro protein purification. They do not contain any genetic material and are therefore safer. However, the low immunogenicity of subunit vaccines and the complex structure and specific physicochemical properties of the mucosal immune system hinder the development of nasal subunit vaccines.

To overcome these barriers, researchers have used different strategies to precisely deliver vaccine antigens to specific mucosal immuno-inducing sites for efficient mucosal immune responses [6, 7].

Advances in nanotechnology used as an antigen delivery system provide new opportunities for overcoming these obstacles and developing intranasal universal influenza vaccines with broad protection against diverse influenza viruses [8–14]. Moreover, compared with soluble vaccines, self-assembled nanoparticles displaying antigens at high density on their surface can be easily recognized by antigen-presenting cells (APCs) to promote immune responses [15]. Dendritic cells (DCs) are considered to be the most potent APCs in vivo. They play an important role in inducing and regulating specific T cell and B cell immune responses [16]. Nanoparticle vaccines have been used in the prevention of infectious diseases such as COVID-19, human immunodeficiency virus (HIV), hepatitis B, influenza, and acquired immunodeficiency syndrome (AIDS) [17–22]. Additionally, fusion of vaccine antigens with mucosal immune cell ligands can achieve targeted delivery of antigens through ligand‒receptor binding, and misfolded cells (M cells) constitute the main gateway for antigens to enter the mucosa‒associated lymphoid tissues of the mucosal immune system. Thus, antigen delivery strategies targeting M cells as a breakthrough point can cause significant mucosal immune responses by inducing secretory IgA and by breaking down existing barriers in the development of mucosal vaccines [23, 24].

Many naturally occurring proteins self-assemble into nanoparticles and can simultaneously display different types of antigenic epitopes on their surfaces, and can be used to prepare multivalent vaccines against a wide range of viral infections [25]. Ferritin is an intracellular iron storage protein that self-assembles into spherical nanocages with a symmetrical structure and an ordered matrix [26]. Ferritin nanoparticles are self-arranged as octahedrons consisting of eight trimeric units, allowing trimeric antigens to be presented in their natural conformation on the surface of the nanoparticles. In addition, ferritin is resistant to heat and chemical stress and is widely used in vaccine development. In the development of HIV vaccines, Sliepen et al. [27] reported that a ferritin nanoparticle vaccine loaded with Env trimers induced more neutralizing antibodies in both mice and rabbits compared with a regular Env trimer vaccine. Joyce et al. [28] developed a ferritin-based SARS-CoV-2 nanoparticle vaccine that was also shown to produce high neutralizing antibody titres. Kanekiyo et al. [19] demonstrated that influenza HA ferritin nanoparticles induced much stronger immune responses than licensed inactivated vaccines and conferred cross-protection against heterologous strains.

Additionally, several studies have identified a 12-mer Co4B ligand which can target conjugated antigens to nasal-associated lymphoid tissue M cells. Intranasally immunized mice with Co4B ligand-conjugated Ag induced not only an efficient antigen-specific mucosal immune response but also a systemic immune response that conferred prominent protective immunity against nasal challenges caused by pathogens in mice [29, 30].

Influenza virus haemagglutinin (HA) is a key protein that mediates viral invasion of host cells and is the primary target of anti-influenza virus-neutralizing antibodies. HA-induced neutralizing antibodies are able to bind to the virus early in the course of infection and inhibit viral invasion of cells [31]. Neutralizing antibodies are the primary factor in antiviral immunity. Therefore, HA has long been the antigenic gene of choice for influenza subunit vaccines. Several HA-based nanoparticle vaccines have been developed and have broadened the protective spectrum [8, 19]. Moreover, the N-terminal ectodomain of influenza A virus matrix protein 2 (M2 ectodomain, M2e) consists of 24 amino acids and is almost completely conserved among all IAVs [32]. Although M2e-specific antibodies do not directly neutralize influenza viruses, M2e-directed antibodies promote the removal of virus-infected lung epithelial cells via antibody-dependent cell cytotoxicity (ADCC) and, to a large extent, provide cross immune-protection, contributing to the reduction in influenza symptoms [33]. Also, because of the low immunogenicity of M2e-based vaccines, combining M2e with other influenza viral antigens to improve protective efficacy can be an ideal strategy [34].

Here, we report the development of an intranasal ferritin-based influenza nanoparticle vaccine that enhances immune responses and broadens the protective spectrum. The ectodomain of HA (H), three repeated M2e epitopes (M), and the M-cell-targeting ligand Co4B (C) were fused in tandem to the N-terminus of ferritin (f). The fusion protein, denoted as CHM-f, was produced through a baculovirus-insect cell system. Intranasal vaccination with CHM-f induced in mice robust humoral and cellular immune responses and conferred complete protection against lethal challenges by divergent influenza viruses. CpG’s are TLR9 receptor agonists that can activate the TLR9 receptor by binding to it. They are considered effective vaccine adjuvants capable of enhancing the strength, breadth, and durability of vaccine responses. In this study, we also evaluated the nanoparticle vaccine’s performance in conjunction with CpG.

## Methods

### Experimental design

The objective of this study is to develop a new generation of influenza vaccines that are safe, efficient and virus-independent and that induce cross-protection. We developed ferritin-based nanoparticle vaccines which were fused with the ectodomain of the influenza HA protein, three sequential repeats of highly conserved M2e epitopes, and the M-cell-targeting ligand Co4B. To validate the immunoenhancing effects of the nanoparticle vaccine, we studied differences in the induction of humoral immune responses and T-cell-mediated cellular immune responses by comparing the nanoparticle vaccine with soluble HA (rHA). We further evaluated the prophylactic protection against both homologous and heterologous influenza strains via intranasal vaccination with the ferritin-based nanoparticle in mice. Statistical analyses were conducted when applicable and are explained in the figure legend. All animal procedures were performed according to the guidelines of Northwest A&F University and approved by the Institutional Animal Care and Use Committee (IACUC).

### Mice, cells and viruses

Female 6-8-week-old specific pathogen-free BALB/c and C57BL/6 mice were purchased from Liaoning Changsheng Biotechnology Co., Ltd. (Liaoning, China). The mice were housed in individually ventilated cage (IVC) systems (Fengshi Group, Suzhou, China) and supplied ad libitum with feed and water.

MDCK cells were cultured in Dulbecco’s modified Eagle’s medium (DMEM, Cytiva, USA) supplemented with 10% fetal bovine serum (FBS, Gibco, USA). DC2.4 cells were cultured in 10% FBS (Gibco, USA) RPMI 1640 medium (Gibco, USA) containing 1% penicillin/streptomycin (Cytiva, USA). Sf9 and Hi5 cells were cultured in IB905 serum-free medium (Yishengke, China).

A/Puerto Rico/8/34 (PR8, H1N1), H3N2 (CVCC AV1520), A/duck/Jiangsu/k1203/2010 (H5N8) and A/Chicken/Jiangsu/7/2002 (H9N2) were cultured in pathogen-free 10-day-old embryonic chicken eggs (Boehringer Ingelheim Witte Biotechnology Co., Ltd., Beijing, China). The 50% lethal dose (LD50) of the viruses was calculated via the Reed & Muench method. The swine virus strain H3N2 (CVCC AV1520) was obtained from the Centers for Veterinary Culture Collection of China (China Institute of Veterinary Drug Control, Beijing, China). The other virus strains were stored in our laboratory.

### Vector construction

The genes encoding the H1N1 (A/victoria/2570/2019 pdm09-like virus) HA protein were downloaded from the GISAID Epiflu database with accession number EPI_ISL_401903. The genes encoding the influenza HA protein were codon optimized for enhanced expression in *Spodoptera frugiperda* (Sf9) insect cells and they were synthesized biochemically by GenScript (Nanjing, China). On this basis, various gene combinations, such as HM and CHM, were constructed via overlapping polymerase chain reaction (PCR). HM fusion genes were generated by fusing three sequential repeats of M2e to the C-terminus of the ectodomain of HA (residues HA1 1–HA2 174) with a Glu-Ala-Ala-Ala-Lys linker. The genes encoding the Co4B peptide were attached to the N-terminus of HM fusion genes via a Gly-Gly-Gly-Gly-Ser linker to generate CHM. All the genes were separately fused to the N-terminus of *Helicobacter pylori* ferritin (residues 5-167, GenBank: NP_223316) via a (G4S)3 linker, and denoted as HA-f, HM-f and CHM-f. And a 6x His tag was added to the C-terminus of ferritin. The soluble HA protein (rHA) in the trimeric form (trimeric HA) contains the full-length HA gene. It is used as a control against nanoparticle vaccines. All the genes mentioned above were cloned and inserted into pBacPAK9 baculovirus transfer vectors between the BamHI- NdeI sites downstream of the polyhedrin promoter.

### Protein expression and purification

To produce rHA, HA-f, HM-f, CHM-f and ferritin-only nanoparticles, the plasmids were co-transfected into Sf9 cells with qBac Bacmid (Shaanxi Bacmid Biotechnology Co., Ltd., Yangling, China) and Sofast Transfection reagent (Sunma, Xiamen, China). Six days after transfection, the recombinant baculovirus (rBV) was passaged to amplify the viral load to the 3rd generation, and the 3rd generation of rBV was used to infect Hi5 cells to express the recombinant protein. Forty-eight hours after infection, the medium supernatant was harvested via centrifugation at 10,000 rpm for 20 min. The target proteins were then concentrated, and the buffer was exchanged with phosphate buffer (25 mM PB, 150 mM NaCl, 0.05% Tween 20, pH 7.0) via cross-flow ultrafiltration.

A two-step purification process was employed to purify the target proteins. Initially, the proteins were purified through size exclusion and binding chromatography using a Capto™ Core 400 column (Cytia, USA), with the effluent collected. The collected effluent underwent further purification via ion-exchange chromatography on an Nuvia™ HP-Q column (Bio-Rad, USA), with the effluent corresponding to each signal peak being collected. After overnight dialysis, the purified proteins were filtered through a 0.22-µm membrane, and the protein concentration was determined with a BCA protein assay kit (Thermo, USA).

### Characterization of the nanoparticles

The purified proteins were verified by reducing sodium dodecyl sulfate–polyacrylamide gel electrophoresis (SDS–PAGE). The morphological features of the nanoparticles were analyzed via transmission electron microscopy. Initially, the purified protein was diluted to 0.2 mg/mL with phosphate buffer. Subsequently, 10 µL of the diluted solution was added dropwise to carbon-coated copper grids. The grids were allowed to dry at room temperature for 2 min, followed by staining with 2% phosphotungstic acid for another 2 min, and the excess liquid was then removed via filter paper. Upon drying, images were recorded on an HT7800 microscope (Hitachi, Japan) at 80 kV.

The particle sizes of the nanoparticles were measured at 25 °C via dynamic light scattering (DLS) using a Zetasizer Nano ZS ZEN3600 (Malvern Instruments Ltd., UK). The purified protein was first diluted to an appropriate concentration in phosphate-buffered saline prior to analysis. The diluted sample was subsequently placed in a plastic cuvette and measured at a fixed scattering angle of 90° for particle size detection.

### Stimulation of DCs by nanoparticles in vitro

Murine bone-marrow-derived DCs (BMDCs) were prepared from bone marrow of 6-8-week-old C57BL/6 and cultured in RPMI 1640 medium (Gibco, USA) supplemented with 10% FBS (Gibco, USA), 1% penicillin/streptomycin (Cytiva, USA), 20 ng/mL of GM-CSF (R&D Systems, USA) and 10 ng/mL of IL-4 (R&D Systems, USA) at 37 °C and 5% CO_2_. Following 6 days of culture, BMDCs were harvested and transferred to 24-well plates (5 × 10^5^ cells/well), and cultured overnight. And BMDCs were treated with LPS (1 µg/mL, Sigma-Aldrich, USA), rHA (5 µg/mL), CHM-f (5 µg/mL) or PBS (10 µL) respectively for another 24 h for further assays.

During the experiment to assess DCs maturation, the antigen-treated BMDCs were collected and washed three times with PBS. Then, the mature BMDCs were stained with fluorescent antibodies, containing CD11c-PerCP-Cy5.5 (BD, USA), CD80-PE (BD USA), and MHC II-FITC (BD, USA), for 30 min at 4 ℃. Following samples being washed three times with PBS, DCs maturation markers were measured with a BD FACSAria™ III flow cytometer.

### Cellular uptake of nanoparticles in vitro

The antigen uptake capacity by cells was evaluated by doing a dextran uptake experiment. 5 × 10^5^/mL antigen-treated mature BMDCs were collected, then centrifuged at 1000 g for 5 minutes, and washed three times with PBS. The cells were incubated with 1 mg/mL FITC-dextran (MW 40 000; Sigma-Aldrich, USA) at 37 °C or4 °C for 2 h. Mean fluorescence intensity (MFI) was measured by flow cytometry. ∆MFI (increase in mean fluorescent intensity) was calculated as follows: ∆MFI = MFI (37 °C treatment) – MFI (4 °C treatment).

To assess the uptake of nanoparticles by cells, CHM-f was labeled using an FITC-conjugation kit (MCE, USA), and NHS-FITC-tagged CHM-f was evaluated by confocal fluorescence imaging. DC2.4 cells were cultured at low density in 35 mm confocal dishes (Biosharp, China) overnight, and FITC-labeled CHM-f was then added (30 µg/well) and incubated for 24 h. Following the incubation, supernatants were gently discarded and the plates washed three times with PBS. Cells were fixed with 4% paraformaldehyde (Biosharp, China) for 15 min, washed 3 times with PBS, and permeabilized with 0.1% Triton X-100/PBS (Biosharp, China) for 5 min. Phalloidin-CoraLite 594 (Proteintech, China) and DAPI (Beyotime, China) were used to stain of F-actin and nuclei, respectively. And confocal images were acquired using an Nikon A1 + A1R + confocal microscope. Fluorescence intensity was measured using Image J software.

### Animal study

Female BALB/c mice (6–8 weeks old) were randomly divided into 10 groups. The mice were intranasally (i.n.) immunized with 50 µL of rHA, HA-f, HM-f or CHM-f in saline (15 µg of H1 protein per mouse). We previously conducted studies on the immune-enhancing effects of different doses of CpG complex adjuvants on vaccines, and the results showed that 20 µg CpG induced the optimal immunity [35]. For the adjuvant groups, all the proteins were mixed with 20 µg of CpG IAMA-002 (JSIAMA, China) before immunization. Additionally, serving as positive controls, mice were intramuscularly (i.m.) immunized with a quadrivalent inactivated influenza vaccine (QIV) containing the same 2019 pdm-like HA at equimolar concentrations of HA. Mice as negative controls were immunized with 50 µL of PBS. The mice were vaccinated twice at an interval of 4 weeks.

Serum samples were collected 3 weeks post-priming and post-boosting immunizations. Nasal and lung washes were performed 3 weeks after immunization and was done by flushing the nostrils or lungs with 1 mL of pre-cooled sterile PBS, followed by centrifugation at 12,000 rpm for 3 min. The spleens of the immunized mice were collected 3 weeks after boosting immunization, and the lymphocytes were harvested using the Mouse 1× Lymphocyte Separation Medium (DAKEWE, China).

Four weeks after immunization, the immunized mice were challenged intranasally (i.n.) with a 15× median lethal dose (LD50) of A/PR/8 (H1N1), a 10× LD50 of H3N2 (CVCC AV1520), a 10× LD50 of A/duck/Jiangsu/k1203/2010 (H5N8) or a 10× LD50 of A/Chicken/Jiangsu/7/2002 (H9N2). Mice were monitored daily for body weight loss and survival rate for 14 days following the challenge. Weight loss greater than 25% of the initial weight was used as the humane endpoint. Five days postinfection, four mice were euthanized, and their lung tissues were harvested for histopathological examination and lung viral titre determination. All viruses and infectious samples were handled in a biosafety level 3 (BSL-3) containment facility except for H1N1 and H3N2, which were handled under BSL-2 laboratory conditions.

### Enzyme-linked immunosorbent assay

Anti-HA/M2e IgG, IgG subclasses and IgA antibody titres were determined via antibody ELISA in sera, BALF and nasal wash samples. Briefly, HA or M2e peptide was coated on 96-well plates at a concentration of 1 µg/mL, 100 µL per well. The plates were then incubated at 4 °C overnight. After washing three times with PBST, the plates were blocked with 5% nonfat milk at 37 °C for 2 h and washed with PBST three times. The immune sera were then serially diluted 2-fold, 100 µL was added to each well of the plates, and the plates were incubated at 37 °C for 1 h. Subsequently, HRP-conjugated goat anti-mouse IgG (Biodragon, China), HRP-conjugated goat anti-mouse IgG1 (Biodragon, China), HRP-conjugated goat anti-mouse IgG2a (Biodragon, China) or HRP-conjugated goat anti-mouse IgA (Proteintech, China) at a 5000-fold dilution was added, and the mixture was incubated at 37 °C for 1 h. Then, 100 µL of 3,3′,5,5′-tetramethylbenzidine solution (TMB, Beyotime, China) was added, the mixture was incubated at 37 °C for 15 min, and the stop solution was then added. The absorbance at 450 nm was measured via a Prolong DNM-9602 Microplate Reader (Pro-long New Technology Co., Ltd., China), and the highest dilution with an OD_450_ value of more than twice that of the PBS group was regarded as the endpoint of the antibody titre.

### HAI and viral neutralization assay

HAI titres of immunized mouse serum samples were determined using 1% chicken erythrocytes and 4 HA units of A/victoria/2570/2019 (IVR-215) antigen. Before performing the HAI assay, the serum samples were treated with receptor destroying enzyme (RDE; Denka Seiken Co., Ltd., Japan) overnight at 37 °C and then heated at 56 °C for 30 min to remove nonspecific inhibitors. Finally, the concentrated chicken erythrocytes were added to RDE-treated sera at a 1:20 volume ratio to remove nonspecific agglutinins. The treated sera were diluted 2-fold serially with saline in 96-well plates, and the highest dilution of serum that inhibited virus haemagglutination was deemed the HAI titre.

A neutralization assay was used to determine the neutralizing antibody titres in the serum. First, the mouse serum samples were inactivated at 56 °C for 30 min, and two-fold serial dilutions were then performed and mixed with 100 × TCID_50_ of influenza virus and incubated at 37 °C for 2 h. Afterward, the mixture was added to MDCK cells (1.5 × 10^5^/mL, 100 µL/well, with 2 µg/mL TPCK-trypsin) in 96-well plates and incubated for 3 days at 37 °C. The neutralization titres were determined by the combination of the cytopathic effect on the cells and the standard haemagglutination assay.

### Flow cytometry analysis

The CD3^+^CD4^+^ and CD3^+^CD8^+^ T-cell subpopulations and intracellular cytokines in the spleen were detected by flow cytometry. The spleens of immunized mice were collected 3 weeks after boost immunization, and lymphocytes were harvested using the Mouse 1× Lymphocyte Separation Medium. The isolated lymphocytes were resuspended in RPMI 1640 medium, transferred to 24-well plates and incubated at 37 °C for 30 min. Then, individual peptide pools (15-mer overlapping by 11 residues, 2.5 µg/mL for each peptide), which cover the HA protein of H1N1, along with 1 µL protein transport inhibitor (BFA, BD, USA), were added to stimulate the cells at 37 °C for 5–6 h. Stimulated lymphocytes were harvested and stained with 50 µL of live/dead stain containing 50 µL of FCS (PBS with 1% FBS) and 1 µL of fixable viability stain 620 (BD, USA) for 15 min at RT and then centrifuged at 600 × g for 5 min, after which the supernatant was discarded. Subsequently, 50 µL of Fc blocker (BD, USA) was added to each sample and incubated at 4 °C for 10 min to eliminate nonspecific binding. After incubation, cell surface staining was performed for 30 min at 4 °C in the dark with APC-Cy7-antimouse-CD3 (BD, USA), PerCP-Cy5.5-antimouse-CD4 (BD, USA), and AmCyan-anti-CD8α antibodies (BD, USA). After cell surface staining, cell fixation and membrane breaking were performed by adding fixation/permeabilization solution (BD, USA), followed by intracellular cytokine staining (ICS) with FITC-conjugated rat anti-mouse IFN-γ (BD), PE-Cy™7-conjugated rat anti-mouse IL-4 (BD), PE-conjugated rat anti-mouse IL-2 (BD), CD154 (CD40 ligand) monoclonal antibodies and eFluor™ 450 (Thermo, USA) and incubation at RT for 40 min. After washing once with 100 µL of 1× Perm Wash solution (BD), the cells were resuspended in 150–200 µL of FCS. The stained cells were tested with a BD FACSAria™ III flow cytometer and analyzed via FlowJo software.

### Histopathological analysis of the lungs

Five days after the challenge, four mice per group were euthanized, and lung tissues were collected from the infected mice. The right lung lobes were fixed in 4% paraformaldehyde overnight at 4 °C and then embedded in paraffin, followed by sectioning and staining with haematoxylin and eosin (H&E). The images of the lung sections were recorded by Chengdu Lilai Biotechnology Co., Ltd.

### Determination of lung virus titres

Lung tissues were homogenized, and the supernatants were obtained via centrifugation at 12,000 rpm for 5 min. Total viral RNA was extracted from the supernatants using a viral genome DNA/RNA extraction kit (TIANGEN, China), after which the RNA was reverse transcribed to cDNA using a reverse transcription kit (TransGen, China). Then, the copy numbers of the viral genome were detected via real-time fluorescence quantitative PCR (qPCR).

### Statistical analysis

All the data were analyzed using GraphPad Prism software (version 8.0.2) and presented as the means ± SEMs. The unpaired t-test was used to estimate the statistical significance of differences between two groups, and one-way ANOVA with Tukey’s multiple comparison test was used to compare the statistical significance between multiple groups. *P* < 0.05 was used to indicate a statistically significant difference.

## Results

### Construction and characterization of the CHM-f nanoparticles

**Fig. 1:**
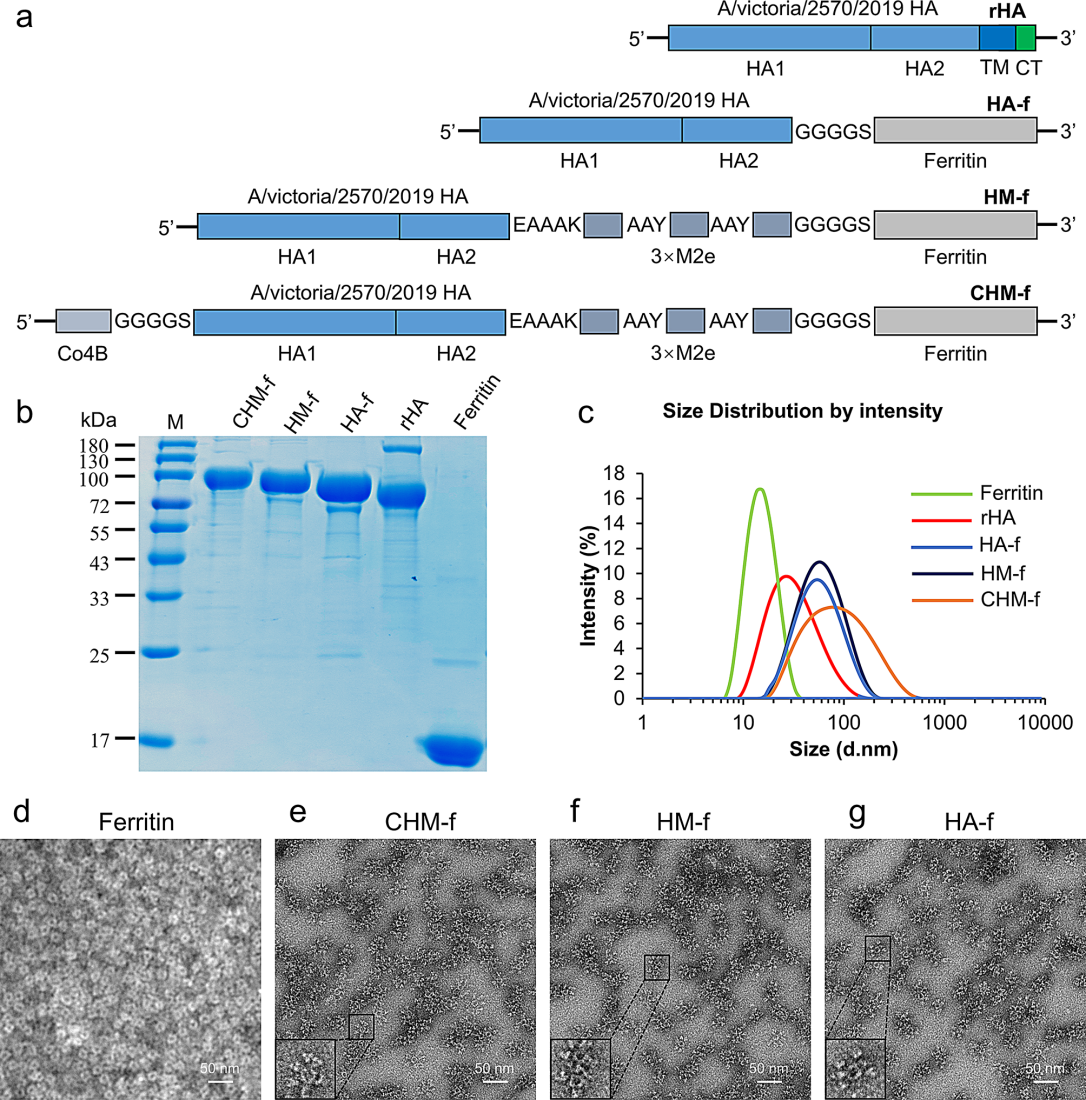
Immunogen design and characterization of nanoparticles. (**a**) schematic diagram of immunogen construction: Plasmid rHA, encoding the HA protein of H1N1, plasmid HA-f, by fusing the ectodomain of the HA protein to the N-terminus of *H. pylori* ferritin; plasmid HM-f, by fusing the ectodomain of the HA protein and three copies of M2e (3M2e) to the N-terminus of ferritin in tandem; and plasmid CHM-f, by fusing the Co4B epitope (C), ectodomain of the HA protein (H), three sequential repeats of M2e epitopes (M) in tandem to the N-terminus of ferritin. (**b**) SDS-PAGE analysis of purified proteins. (**c**) Dynamic light scattering of nanoparticles HA-f, HM-f and CHM-f. (**d**) TEM images of assembled ferritin. (**e**) TEM images of assembled CHM-f nanoparticles. (**f**) TEM images of assembled HM-f nanoparticles. (**g**) TEM images of assembled HA-f nanoparticles

To construct the nanoparticles, the ectodomain of the A/victoria/2570/2019 pdm09-like virus HA protein was fused to the N-terminus of *Helicobacter pylori* ferritin, individually or in tandem with different epitopes (Fig. 1a). For example, CHM-f nanoparticles were constructed by fusing the Co4B epitope (C), HA antigen (H), and three sequential repeats of M2e epitopes (M) in tandem to the N-terminus of *H. pylori* ferritin (Fig. 1a). Other fusion proteins (HA-f, HM-f), soluble HA protein (rHA), and ferritin alone were generated as controls. After expression in insect cells using baculovirus, all the proteins were purified by size exclusion, binding chromatography and ion-exchange chromatography. The molecular size of the fusion proteins was determined via SDS‒PAGE. The results revealed a major band between 70 and 100 kDa for rHA and between 100 and 130 kDa for nanoparticles (Fig. 1b). The purified nanoparticles were also examined by dynamic light scattering (DLS), which revealed that the particle size ranged from 50 to 60 nm in diameter, whereas that of ferritin alone was approximately 15 nm (Fig. 1c). Transmission electron microscopy (TEM) images revealed that ferritin alone formed homogeneous cage-like particles and the nanoparticles displayed visible spikes around the particles (Fig. 1d-g). Moreover, assessment of nanoparticle stability under different storage temperatures showed that the CHM-f nanoparticle vaccine retains activity and remains stable without discernible degradation, and the assembly of nanoparticles significantly improved the structural stability of the HA antigen (Fig. S1).

### Endocytosis of nanoparticles by mouse DCs

**Fig. 2:**
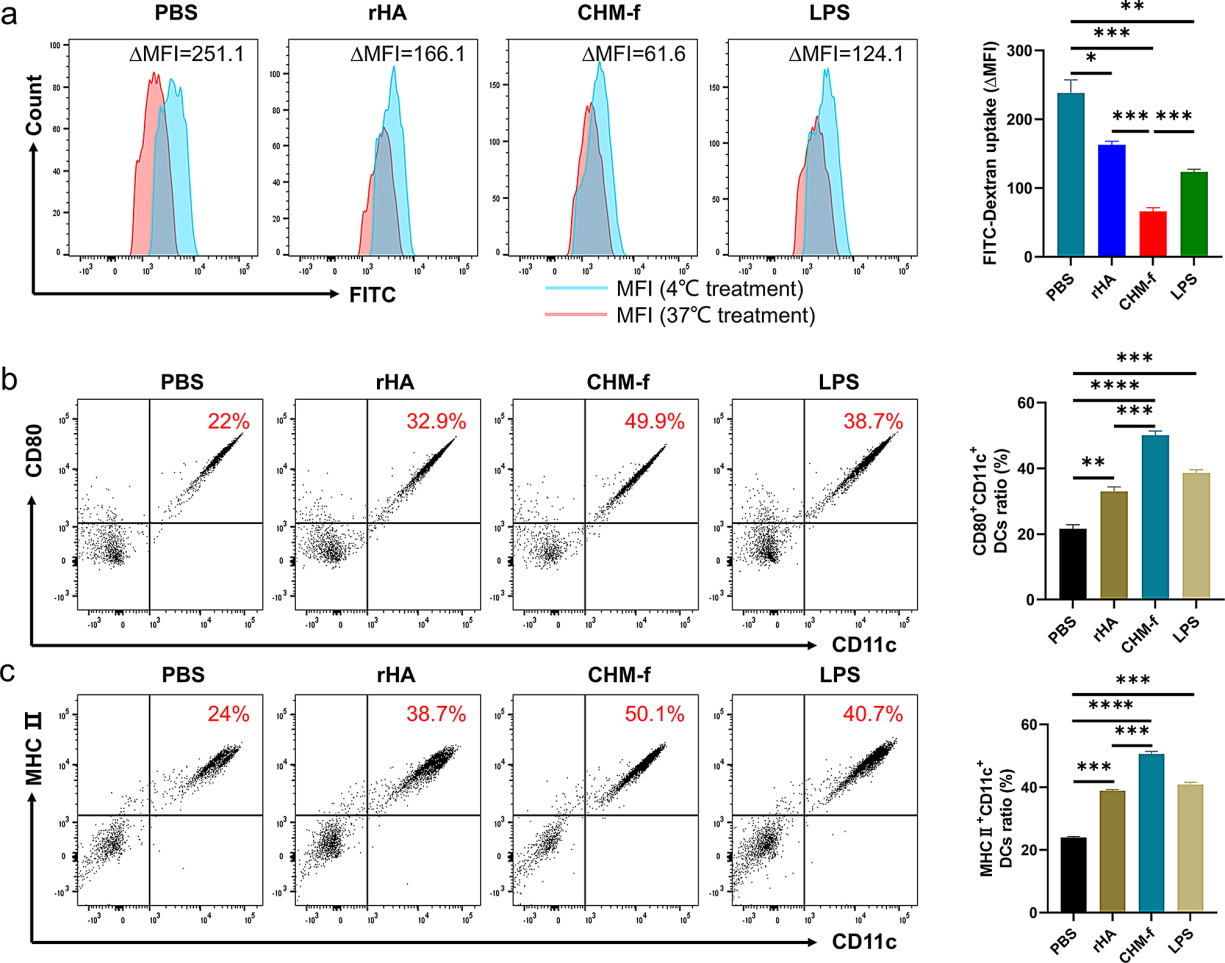
CHM-f elicited BMDCs maturation in vitro. (**a**) PBS-, rHA-, CHM-f- or LPS treated BMDCs were stained with FITC-dextran at 37 ℃ and at 4 ℃ (as negative controls) and analyzed with flow cytometry. (**b**) Flow cytometry analysis of the surface molecule CD80. (**c**) Flow cytometry analysis of the surface molecule MHC II. The data are expressed as the means ± SEMs (*n* = 4, *P < 0.05, **P < 0.01, ***P < 0.001, ****P < 0.0001, ns, not significant)

Immature DCs (imDCs) have an extremely high capacity for antigen phagocytosis. However, following stimulation with antigens, imDCs mature and their antigen uptake capacity is significantly decreased and they differentiate into immunostimulatory APCs [36]. Here, imDCs were stimulated with equivalent doses of rHA and CHM-f, LPS was used as control, and then stained with FITC-dextran to analyze their antigen uptake by flow cytometry. The ΔMFI values of these cells were 166.1, 61.6, and 124.1, respectively for rHA and CHM-f, LPS, whereas the ΔMFI value of PBS-treated DCs was 251.1 (Fig. 2a). These values indicated that CHM-f-treated imDCs significantly inhibited the endocytosis ability of DCs, demonstrating that CHM-f nanoparticles induced DCs maturation.

The antigen presentation process from DCs to naïve T cells requires the assistance of MHC-II and the co-stimulatory molecules CD80 and CD86 [37]. To explore whether CHM-f nanoparticles influence the expression levels of the co-stimulatory molecules by DCs, the percentage of double positive cells for MHC-II/CD11c or CD80/CD11C were analyzed. Like LPS-treated DCs, CHM-f-treated DCs exhibited higher levels of MHC-II and CD80 than rHA- and PBS-treated DCs (Fig. 2b and c). These results showed that CHM-f nanoparticles upregulate the expression of MHC-II and CD80 molecules on DCs, which are essential for T-cell activation.

**Fig. 3:**
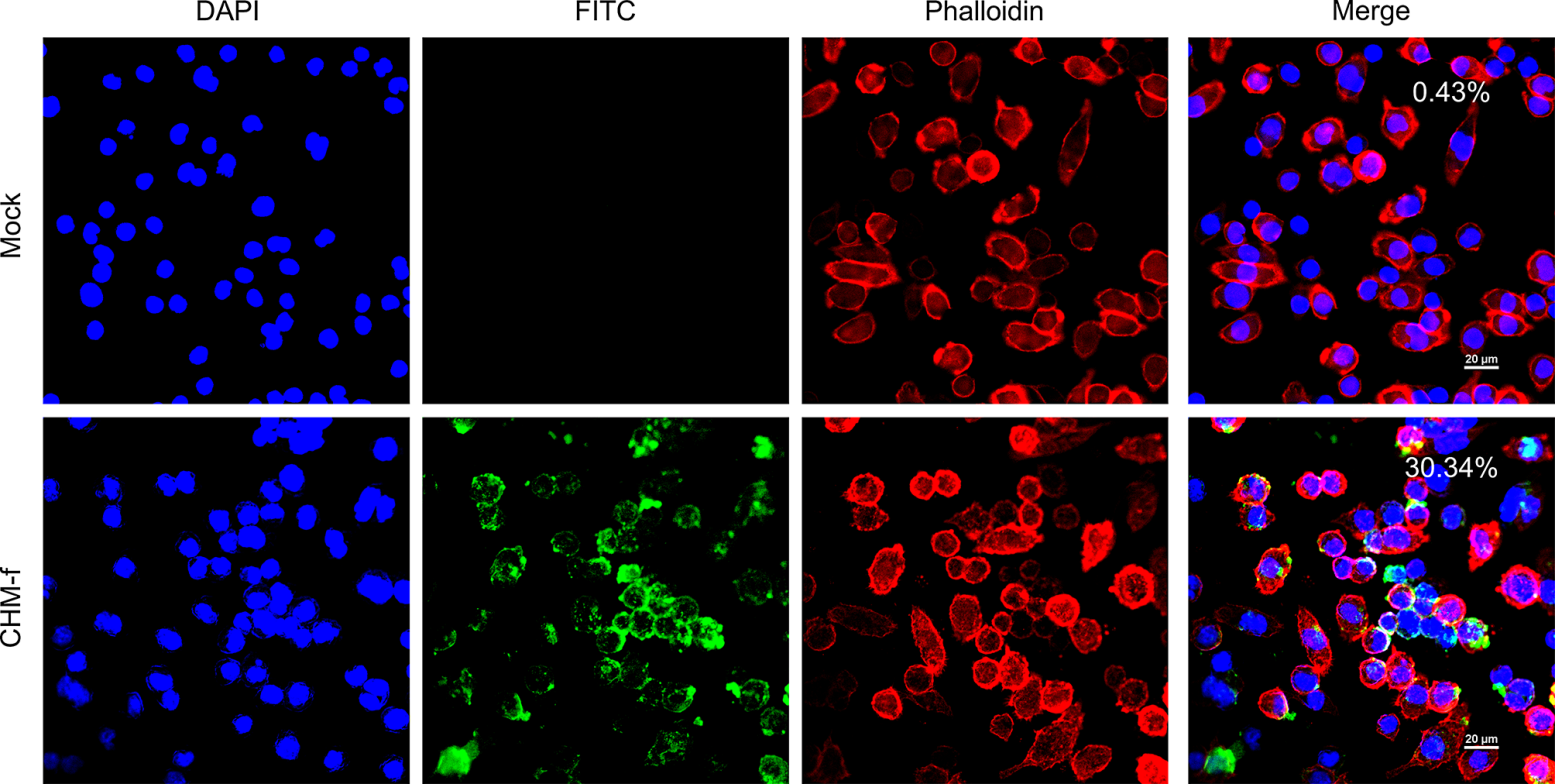
Confocal images of cellular uptake of CHM-f. Visualization of cellular uptake of FITC-labeled CHM-f-treated DCs after 24 h. The blue staining indicates DAPI-stained nuclei. The red staining indicates phalloidin-CoraLite 594-stained actin cytoskeleton. Free FITC was used as a control

Internalization of antigens is a crucial prerequisite for the activation of dendritic cells (DCs) and subsequent antigen cross-presentation. To assess whether the CHM-f can be recognized and processed by the host immune system, we conducted cellular uptake experiments in vitro. FITC-labeled CHM-f was co-cultured with mouse dendritic cells (DC2.4) for 24 h. Laser scanning confocal microscopy showed that in comparison to the mock treated cells, the fluorescence signals associated with the cells treated with CHM-f were significantly enhanced, and the green fluorescence was mainly distributed in the cytoplasm (Fig. 3). These findings suggest that DCs can efficiently take up and internalize CHM-f. These results showed that the nanoparticles can be effectively phagocytosed by DCs, potentiating the antigen presentation.

### Induction of humoral immune responses by CHM-f nanoparticles in mice

**Fig. 4:**
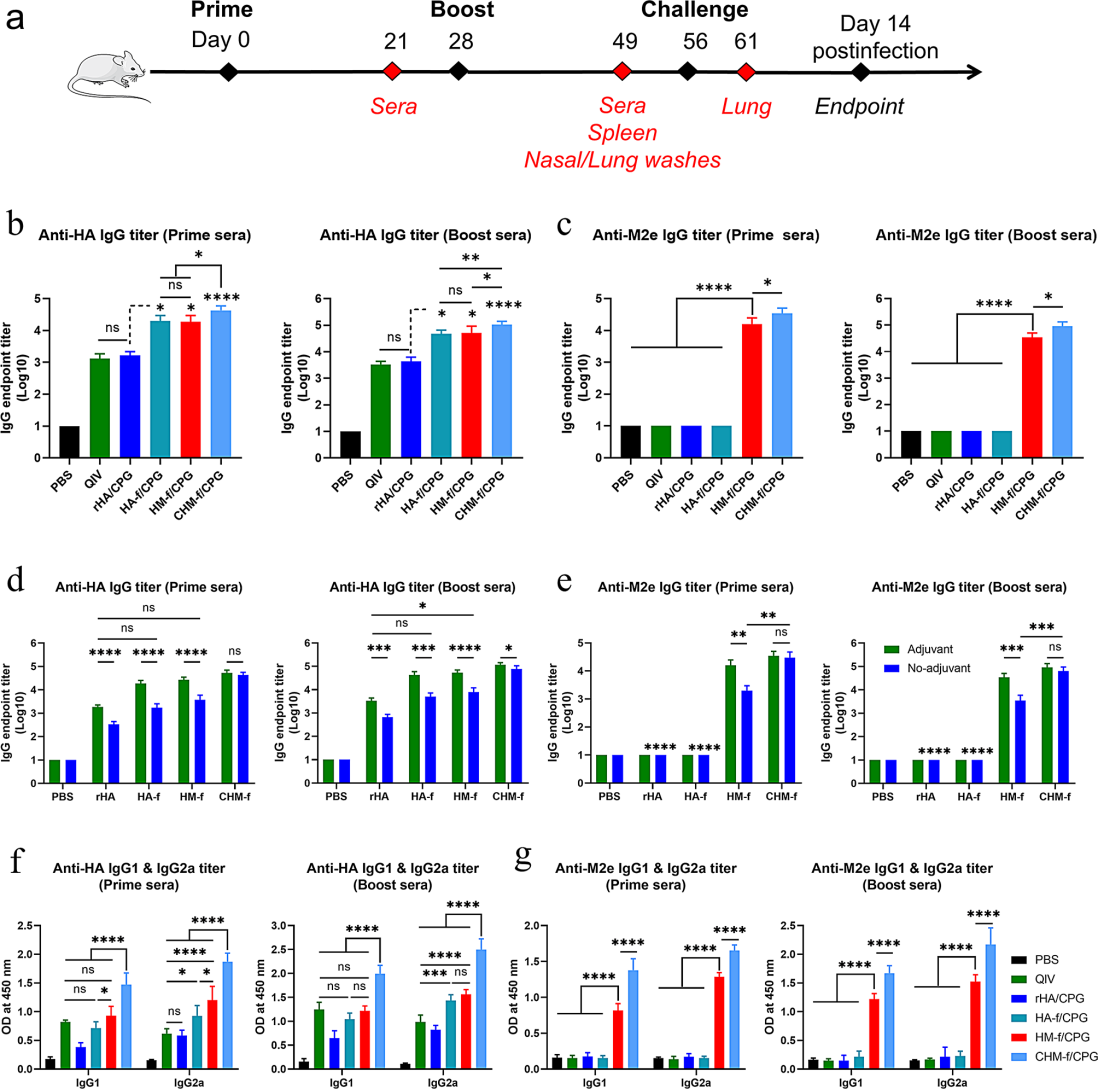
Evaluation of the immunity and production of antigen-specific IgG in CHM-f nanoparticle-immunized mice. (**a**) Schematic of immunization, sample collection, and challenge experiments. (**b-c**) HA- and M2e-specific IgG endpoint titres in prime and boost sera of the adjuvant groups were determined via ELISA. (**d-e**) HA- and M2e-specific IgG endpoint titres in prime and boost sera of the no-adjuvant groups compared with those of the adjuvant groups, **d** and **e** share a common legend. (**f-g**) Anti-HA and M2e-specific IgG1 and IgG2a titres in prime and boost sera, **f** and **g** share a common legend. The data are expressed as the means ± SEMs (*n* = 6, *P < 0.05, **P < 0.01, ***P < 0.001, ****P < 0.0001, ns, not significant)

To investigate the immunogenicity of the nanoparticles, we randomly divided the BALB/c mice into 10 groups, and eight of them were intranasally (i.n.) immunized with 15 µg of rHA, HA-f, HM-f or CHM-f with or without 20 µg of CpG IAMA-002. The mice were monitored daily for potential adverse effects. Their body weight was measured for one week after vaccination, and no significant changes were detected. Seven days after immunization, we collected the lungs of mice for pathological analysis, which showed no significant pathological changes in the lungs (Fig. S2). Mice intramuscularly (i.m.) immunized with quadrivalent inactivated influenza vaccine (QIV) served as the positive control group, and intranasally (i.n.) immunized with PBS served as the negative control group. All mice were immunized at week 0 and given a booster immunization at week 4 (Fig. 4a).

Compared with the rHA/CpG and QIV groups, all the nanoparticle groups (HA-f/CpG, HM-f/CpG, and CHM-f/CpG) elicited significantly higher HA-specific IgG titres in both prime sera (*P* < 0.05, *P* < 0.05, *P* < 0.0001) and boost sera (*P* < 0.05, *P* < 0.05, *P* < 0.0001), and there was no significant difference between the rHA/CpG and QIV groups (Fig. 4b). As shown in Fig. 4b, the high HA-specific IgG titres, which were greater than 10^5^, were induced in the nanoparticle groups following boost immunization. The CHM-f/CpG group presented higher HA-specific IgG titres than the HA-f/CpG (*P* < 0.01) and HM-f/CpG (*P* < 0.01) groups, while the difference was not significant between the HA-f/CpG and HM-f/CpG groups. As shown in Fig. 4c, the HM-f/CpG and CHM-f/CpG groups induced notably greater M2e-specific IgG titres compared with the other groups (*P* < 0.0001), and the M2e-specific IgG antibody levels in the CHM-f/CpG nanoparticle group were greater than those in the HM-f/CpG nanoparticle group (*P* < 0.01). For the no-adjuvant groups, the results demonstrated that all groups except CHM-f presented significantly lower HA-specific IgG titres than the adjuvant groups (Fig. 4d). As shown in Fig. 4d, the CHM-f group presented higher levels of HA-specific IgG antibodies than the rHA (*P* < 0.0001), HA-f (*P* < 0.0001), and HM-f (*P* < 0.0001) groups. Notably, the HA-f and HM-f groups in the absence of adjuvant induced IgG antibody levels comparable to those in the rHA group with adjuvant. Additionally, the CHM-f adjuvant and no-adjuvant groups induced comparable levels of IgG antibodies. As shown in Fig. 4e, lower M2e-specific IgG titres were induced in the HM-f no-adjuvant group than in the adjuvant group in both the prime serum (*P* < 0.01) and boost serum (*P* < 0.001) samples. However, for the CHM-f groups, there was no significant difference between the adjuvant and no-adjuvant groups. These results suggested that CHM-f nanoparticles may have a better immune effect among these ferritin-based nanoparticle vaccines.

For a more detailed evaluation of the immune response, we further examined the IgG subclasses of the induced antibodies. The findings revealed that the nanoparticle groups induced more significant HA-specific IgG1 and IgG2a antibodies compared with the rHA/CpG group in both prime sera and boost sera (Fig. 4f). Among them, the immune effect of CHM-f/CpG was particularly obvious, with higher levels of HA-specific IgG1 and IgG2a than those in the other groups (*P* < 0.0001). The number of M2e-specific IgG antibody subtypes induced by CHM-f/CPG and HM-f/CPG was notably greater than that in the other groups (*P* < 0.0001) (Fig. 4g). Compared with those in the HM-f/CpG group (*P* < 0.0001), the M2e-specific IgG antibody subtype titres were significantly elevated in the CHM-f/CpG group (Fig. 4g). Antigen-specific IgG1 and IgG2a titres in serum are commonly used to assess whether the vaccine-induced immune response is biased toward Th2 or Th1. The IgG1 response elicited by the QIV group was stronger than that elicited by the IgG2a group, indicating that the QIV group was biased toward the Th2 immune response. However, the rHA/CPG and nanoparticle groups induced a Th1-favored immune response, which was characterized by higher levels of IgG2a antibodies than IgG1 antibodies.

**Fig. 5:**
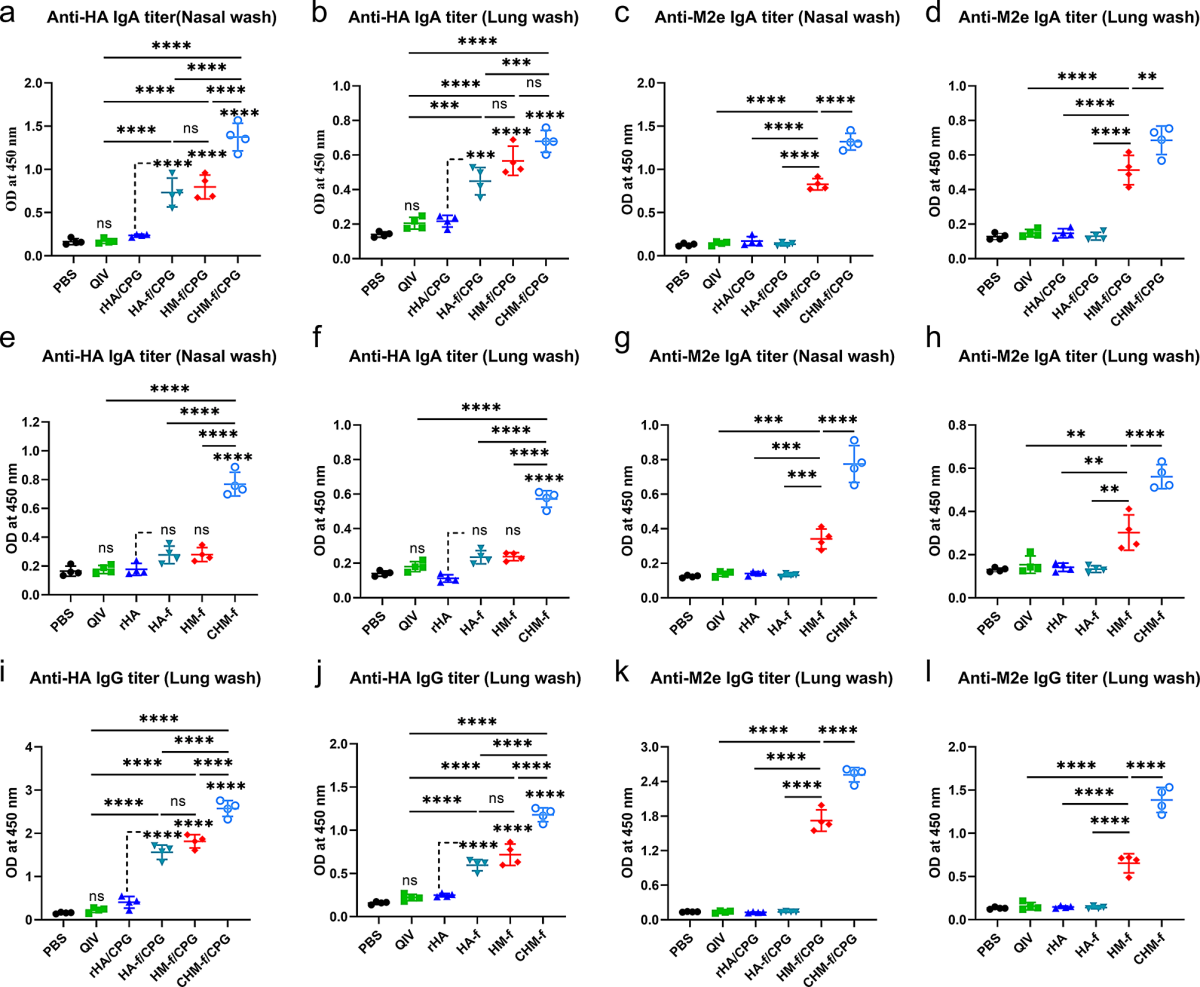
Induction of local mucosal immune responses by immunization with CHM-f nanoparticles in mice. (**a-l**) HA- and M2e-specific antibody levels were measured by ELISA and are presented as OD450 nm values. (**a-b**) Anti-HA IgA levels in nasal and lung washes from the adjuvant groups. (**c-d**) Anti-M2e IgA levels in nasal and lung washes from the adjuvant groups. (**e-f**) Anti-HA IgA levels in nasal and lung washes from the no-adjuvant groups. (**g-h**) Anti-M2e IgA levels in nasal and lung washes from the no-adjuvant groups. (**i-j**) Anti-HA IgG levels in lung washes from the adjuvant and no-adjuvant groups. (**k-l**) Anti-M2e IgG levels in lung washes from the adjuvant and no-adjuvant groups. The data are expressed as the means ± SEMs (*n* = 4, **P < 0.01, ***P < 0.001, ****P < 0.0001, ns, not significant)

To evaluate the immune responses induced by intranasal immunization in the respiratory mucosa, the mucosal immunoglobulin A (IgA) and IgG titres were titrated from nasal and lung wash samples, which were collected 21 days after boost immunization. In the undiluted nasal washes, the HA- and M2e-specific sIgA levels induced by CHM-f/CpG were notably greater than those in the other groups (Fig. 5a and c). Compared with those in the QIV and rHA/CPG groups, the level of sIgA in the HA-f/CpG and HM-f/CpG groups also increased (Fig. 5a and c). Even in the absence of the CpG adjuvant, CHM-f also resulted in significantly elevated titres of sIgA than the other groups (Fig. 5e and g), although CPG adjuvant group showed higher IgA titre. However, the IgG titres elicited by all the groups were especially low in nasal washes (data not shown). In the undiluted lung washes, significantly higher HA and M2e-specific IgG titres were elicited in the HA-f/CpG (*P* < 0.0001), HM-f/CpG (*P* < 0.0001), and CHM-f/CpG (*P* < 0.0001) groups than in the QIV and rHA/CpG groups (Fig. 5i and k). As shown in Fig. 5b and d, in contrast to those in the QIV and rHA/CPG groups, the HA- and M2e-specific sIgA antibody levels were elevated in all the nanoparticle groups. Among them, the CHM-f/CpG group induced the highest levels of antigen-specific antibodies. In the absence of the CpG adjuvant, the CHM-f group was still able to induce higher levels of HA- and M2e-specific IgG and IgA antibodies compared with the other groups (Fig. 5f, h, j and l). Two months after booster immunization, we euthanized the remaining three mice in each group and collected nasal and lung washes for the detection of IgA antibody levels. Importantly, the results showed that the IgA antibody levels of the mice in CHM-f/CpG group remained at a high level two months after booster immunization (Fig. S3). The respiratory mucosal antibody levels were consistent with those in previous studies showing that sIgA and IgG antibodies dominated the upper and lower respiratory tracts, respectively [38].

**Fig. 6:**
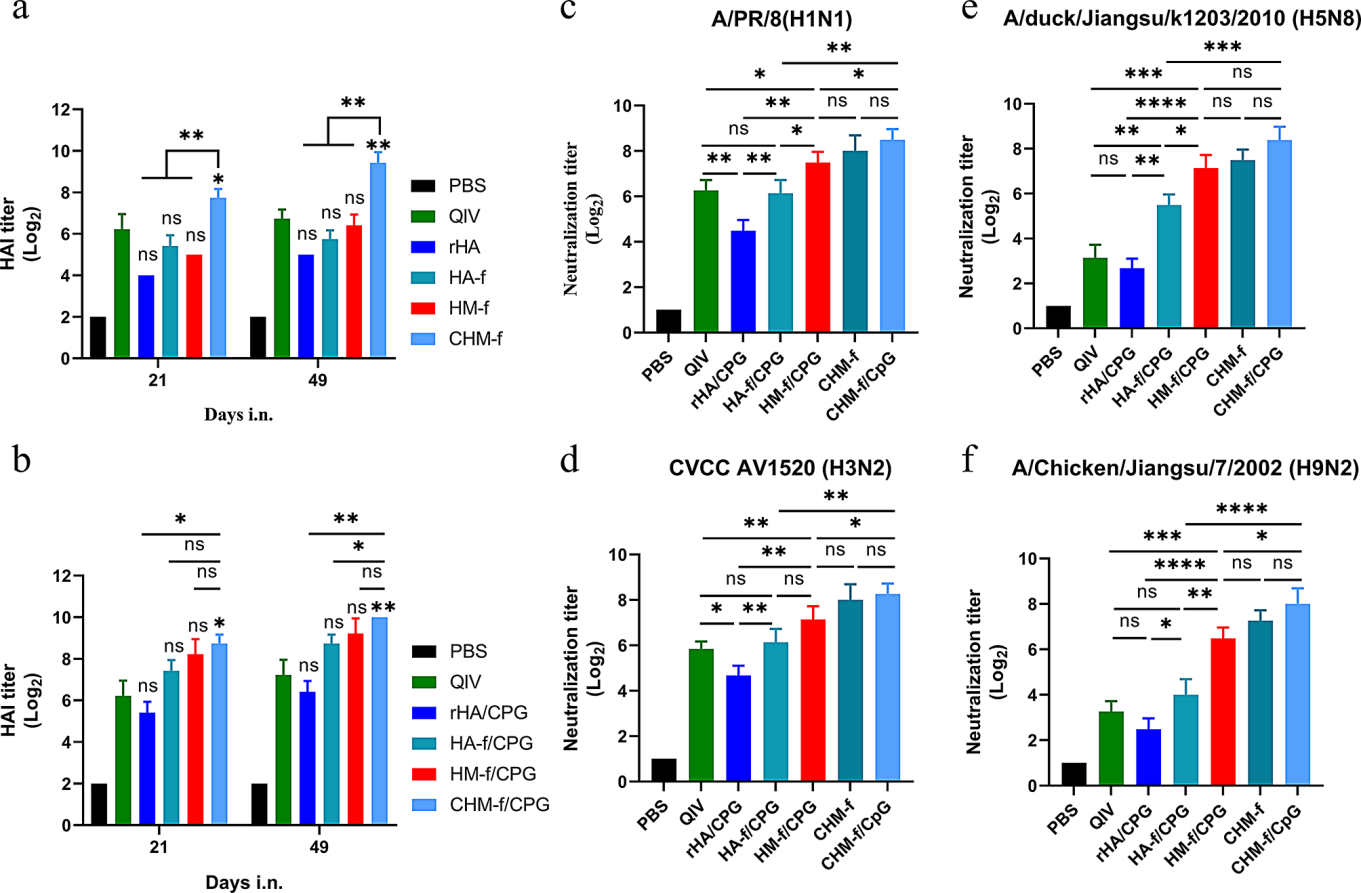
Induction of HAI and neutralizing antibody (nAb) titres in mice immunized with CHM-f nanoparticles. (**a-b**) HAI titres in prime and boost sera of the adjuvant and no-adjuvant groups. (**c-f**) Neutralization titres in boost sera. (**c**) Anti-H1N1 nAb titres. (**d**) Anti-H3N2 nAb titres. (**e**) Anti-H5N8 nAb titres. (**f**) Anti-H9N2 nAb titres. The data are expressed as the means ± SEMs (*n* = 5, *P < 0.05, **P < 0.01, ***P < 0.001, ****P < 0.0001, ns, not significant)

In influenza infections, a haemagglutination-inhibition (HAI) titre of 1:40 is thought to provide 50% protection against influenza infection [39], although estimates range from 1:17 to 1:110 in the literature [40, 41]. Our HAI titre results revealed that the CHM-f without CpG group presented 2.9-, 13.3-, 5.0- and 6.7-fold higher HAI titres compared with the QIV (*P* < 0.05), rHA (*P* < 0.01), HA-f (*P* < 0.01) and HM-f (*P* < 0.01) groups at 21 days postimmunization respectively (Fig. 6a). The HAI titres increased by up to 6.4-, 21.3-, 12.8-, and 8.0-fold at 21 days following boost immunization (*P* < 0.01) (Fig. 6b). Additionally, the HAI titre was greater in the QIV group than in the rHA, HA-f, and HM-f groups, but this difference did not reach statistical significance (Fig. 6a). However, when the CPG adjuvant was used, the HAI titre was greater in all the nanoparticle groups than in the QIV group. As shown in Fig. 6b, the HAI titres in the HA-f/CpG, HM-f/CpG and CHM-f/CpG (*P* < 0.05) groups were 2.3-, 4.0- and 5.7-fold greater than those in the QIV group at 21 days postimmunization respectively. After boost immunization, the HAI titres were 2.8-, 4.0- and 6.9-fold greater in the HA-f/CpG, HM-f/CpG and CHM-f/CpG groups (*P* < 0.01) than in the QIV group respectively. However, the increase induced by HA-f/CpG or HM-f/CpG did not reach statistical significance.

Cross-neutralization and protection against homo/heteroviruses by antibodies produced after boost immunization were evaluated. Neutralizing antibodies have antiviral effects and are powerful weapons in the fight against viral infections. To assess the titres of cross-neutralizing antibodies in the serum at 21 days after boost immunization, four different influenza strains were chosen: A/Puerto Rico/8/34 (PR8, H1N1), H3N2 (CVCC AV1520), A/duck/Jiangsu/k1203/2010 (H5N8), and A/Chicken/Jiangsu/7/2002 (H9N2). As shown in Fig. 6c-f, the rHA/CpG group presented low titres of neutralizing antibodies. In contrast, the HM-f/CpG and CHM-f/CpG nanoparticle groups presented elevated titres of specific neutralizing antibodies against the four strains. A greater number of specific neutralizing antibodies against homologous strains of H1N1 and H3N2 were induced in the QIV group than in the rHA/CpG group (Fig. 6c and d), but low levels of cross-neutralizing antibodies against heterologous strains of H5N8 and H9N2 were detected (Fig. 6e and f). Importantly, the CHM-f/CpG nanoparticle group induced the highest levels of neutralizing antibodies, and even without CpG adjuvant, the CHM-f nanoparticle group induced neutralizing antibody titres comparable to those of the CHM-f/CpG nanoparticle group.

CpG is a mucosal immune adjuvant that induces strong mucosal immune responses [42–45]. In this study, Fig. 5 shows IgG and IgA antibody levels in mouse nasal and lung washes, and CHM-f/CpG generated significantly higher IgG and IgA responses compared to CHM-f. The results confirmed that CpG could induce robust mucosal immune responses. Figure 4 shows specific IgG antibody levels in mouse serum, and the IgG antibody titres of CHM-f/CpG were slightly higher than CHM-f. We also tested the neutralizing antibody titres in the serum of mice, and the results showed that the neutralizing antibody titres of CHM-f/CpG were comparable or slightly higher than CHM-f, which is consistent with the results in Fig. 4. Antigens introduced via the mucosal immunization route are initially captured at mucosal sites by M cells and dendritic cells (DCs) and presented to local T and B cells. These lymphocytes are activated and begin to proliferate within mucosa-associated lymphoid tissue (MALT). A portion of the mature effector T and B cells subsequently migrates into the peripheral circulatory system, and these mucosa-derived effector lymphocytes can further proliferate within peripheral lymph nodes, secreting cytokines and antibodies, thereby enhancing the systemic immune response. Because there may be some bias in the migration of mucosal antibody responses to the peripheral circulatory system, CpG exerted a good humoral immune-enhancing effect at mucosal immune system, but the role played by CpG in the peripheral circulatory system is not obvious.

Taken together, the CHM-f nanoparticles induced not only strong systemic but also local mucosal immunity. Without the CpG adjuvant, the CHM-f group also induced robust antigen-specific antibody responses, indicating that the Co4B epitope along with ferritin nanoparticles can act as an adjuvant to enhance the immune response.

### Induction of cellular immune responses by CHM-f nanoparticles in mice

Rapid T-cell-mediated immune response following vaccination plays a crucial role in the prophylactic and protective phases of vaccines. To further determine whether nanoparticles can induce a robust T-cell response, we isolated splenic lymphocytes from mice 21 days after boost immunization to detect T-cell immune responses via flow cytometry. Gating strategies were used for effective flow cytometry data analysis of T lymphocytes (Fig. S4).

**Fig. 7:**
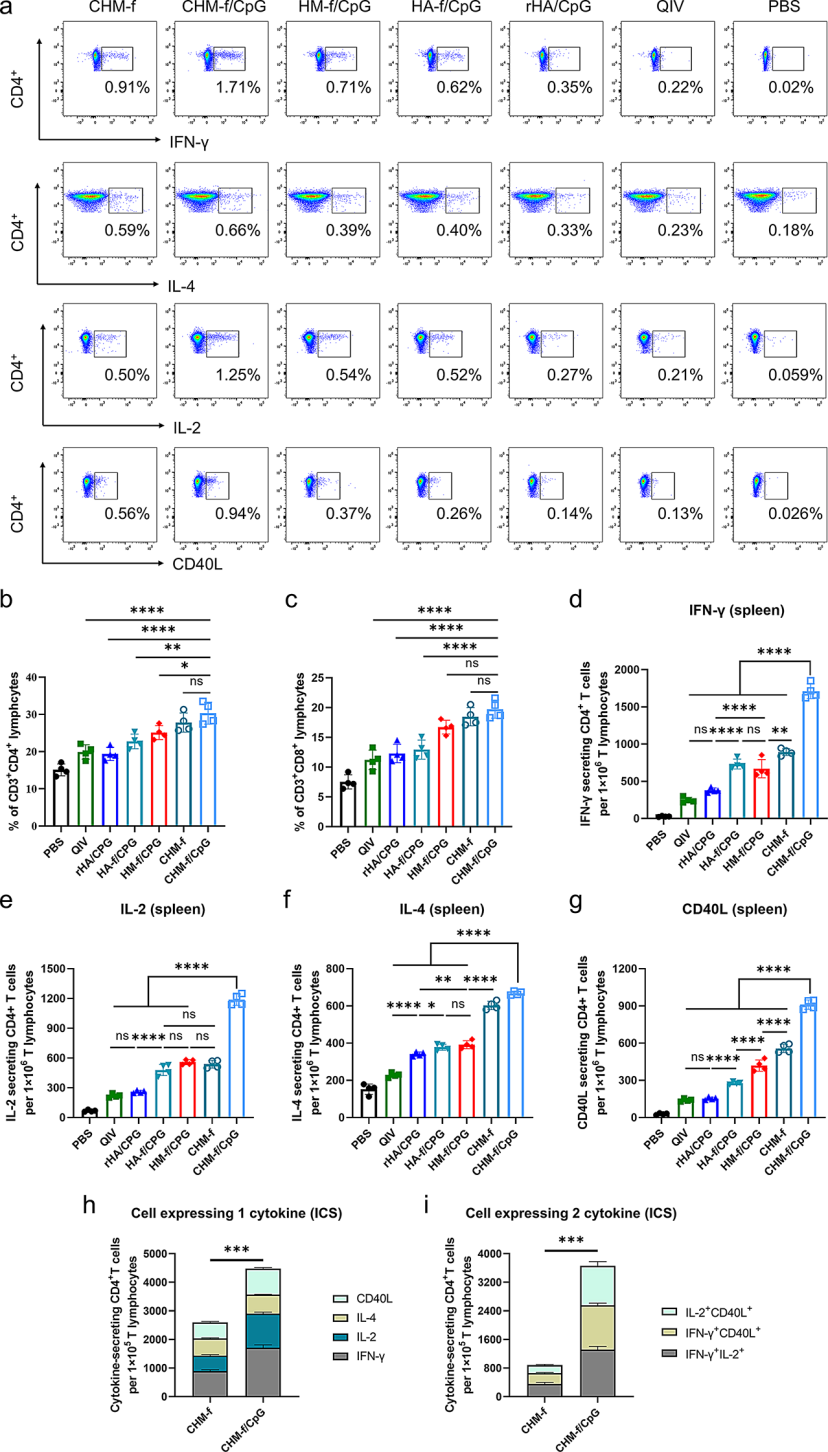
Cellular immune responses. (**a**) Representative flow cytometry analysis results of the percentage of cytokine-secreting CD4^+^ T cells among splenic lymphocytes. (**b**) Percentage of CD3^+^CD4^+^ T cells in splenic lymphocytes. (**c**) Percentage of CD3^+^CD8^+^ T cells in splenic lymphocytes. (**d-g**) The number of intracellular cytokines in splenic CD4^+^ T cells. (**d**) Number of IFN-γ-secreting CD4^+^ T cells. (**e**) Number of IL-2-secreting CD4^+^ T cells. (**f**) Number of IL-4-secreting CD4^+^ T cells. (**g**) Number of CD40L-secreting CD4^+^ T cells. (**h**) Number of single cytokine-secreting CD4^+ ^T cells. (**i**) Number of double cytokine-secreting CD4^+ ^T cells. The data are expressed as the means ± SEMs (*n* = 4, *P < 0.05, **P < 0.01, ***P < 0.001, ****P < 0.0001, ns, not significant)

The representative flow cytometry analysis results of the percentage of cytokine-secreting CD4^+^ T cells, CD3^+^CD4^+^ and CD3^+^CD8^+^ T cells among splenic lymphocytes are shown in Fig. 7a and S5. The results demonstrated that all immunization groups presented higher CD4^+^ and CD8^+^ T-cell rates compared with the PBS control group. The percentages of CD4^+^ and CD8^+^ T cells were significantly increased in the CHM-f/CpG nanoparticle group. Even in the absence of the CpG adjuvant, the CHM-f nanoparticle group still presented higher levels of CD4^+^ and CD8^+^ T cells than the other groups, and no significant difference between the CHM-f/CpG and CHM-f nanoparticle groups was found (Fig. 7b and c).

Intracellular cytokine expression levels of IFN-γ (Fig. 7d), IL-2 (Fig. 7e), IL-4 (Fig. 7f), and CD40L (Fig. 7g) in spleen CD4^+^ T cells, which are indicators of Th1- and Th2-biased immune responses, were detected via intracellular cytokine staining combined with flow cytometry (Fig. 7a). Compared with those in the other groups, the levels of IFN-γ (*P* < 0.0001), IL-4 (*P* < 0.0001), IL-2 (*P* < 0.0001) and CD40L (*P* < 0.0001) in the mice immunized with CHM-f/CpG nanoparticles significantly increased. Compared with those in the rHA/CpG and QIV groups, the expression levels of all four cytokines were notably elevated in the splenic lymphocytes of the mice in the nanoparticle groups. We also observed significantly greater numbers of IFN-γ-, IL-4- or CD40L-secreting CD4^+^ T cells in the CHM-f no-adjuvant group than in the other groups (Fig. 7d, e and g). However, we found no significant difference in the number of IL-2-secreting CD4^+^ T cells among the CHM-f, HM-f/CpG and HA-f/CpG groups (Fig. 7f). CHM-f/CpG also induced a significantly higher percentage of HA-specific CD4^+^ T cells that secrete single or double cytokines, compared to those induced by CHM-f (Fig. 7h and i).

These findings demonstrated that nanoparticles can elicit potent T-cell immune responses. Importantly, bi-epitopic HA + M2e nanoparticles induced greater T-cell immune responses than mono-epitopic HA nanoparticles. Especially CHM-f, which combines HA, M2e and Co4B, induced the greatest number of cellular immune responses. The addition of CpG adjuvant can enhance the levels of IFN-γ, IL-2 and CD40L expressing T cells.

### Intranasal vaccination with CHM-f nanoparticles provides cross-protection

**Fig. 8:**
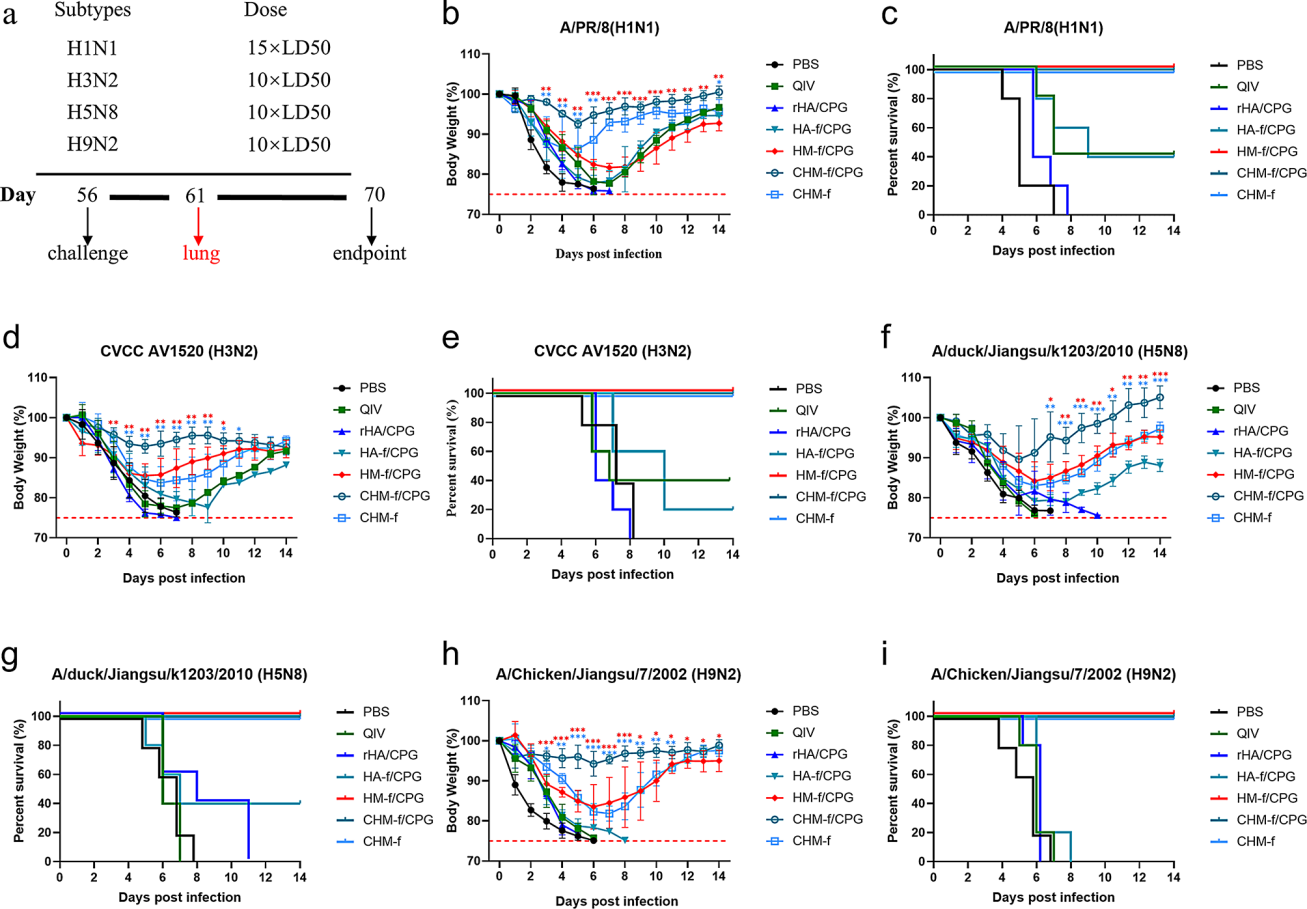
Protective efficacy against homologous and heterologous influenza virus lethal challenges. (**a**) Experimental schema. (**b-i**) Body weight changes and survival rates of the mice after challenge with different influenza viruses for 14 days postinfection; *n* = 5. (**b-c**) 15×LD50 of A/PR/8 (H1N1). (**d-e**) 10× LD50 of H3N2 (CVCC AV1520). (**f-g**) 10× LD50 of A/duck/Jiangsu/k1203/2010 (H5N8). (**h-i**) 10× LD50 of A/Chicken/Jiangsu/7/2002 (H9N2). The data are expressed as the means ± SEMs (*P < 0.05, **P < 0.01, ***P < 0.001)

To evaluate the cross-protective effects of different immunization groups, the mice were immunized twice at 4-week intervals. Four weeks after the boost immunization, the immunized mice were challenged with influenza strains (Fig. 8b-i), including a 15 × median lethal dose (LD50) of A/PR/8 (H1N1), a 10 × LD50 of H3N2 (CVCC AV1520), a 10 × LD50 of A/duck/Jiangsu/k1203/2010 (H5N8) or a 10 × LD50 of A/Chicken/Jiangsu/7/2002 (H9N2). Following the challenge, the body weight changes and survival rates of the mice were monitored continuously for 14 days. As shown in Fig. 8b-i, mice in the CHM-f/CpG immunization group survived all of the challenges with slight weight loss and recovered quickly. In contrast, the mice in the PBS and rHA/CpG groups experienced severe weight loss (over 25%) and died within 9 days. The mice in the CHM-f and HM-f/CpG immunization groups also survived all the challenges but experienced greater weight loss than the CHM-f/CpG-immunized mice and eventually recovered. HA-f/CpG immunization provided partial protection (20-40% mouse survival) with severe body weight loss when the mice were challenged with four different influenza strains. QIV immunization provided only 40% partial protection against H1N1 and H3N2 (Fig. 8b-e), and no protection against heterologous strains of H5N8 and H9N2 (Fig. 8f-i). These results demonstrated that CHM-f nanoparticles can provide excellent cross-protection against lethal challenges caused by various influenza strains.

**Fig. 9:**
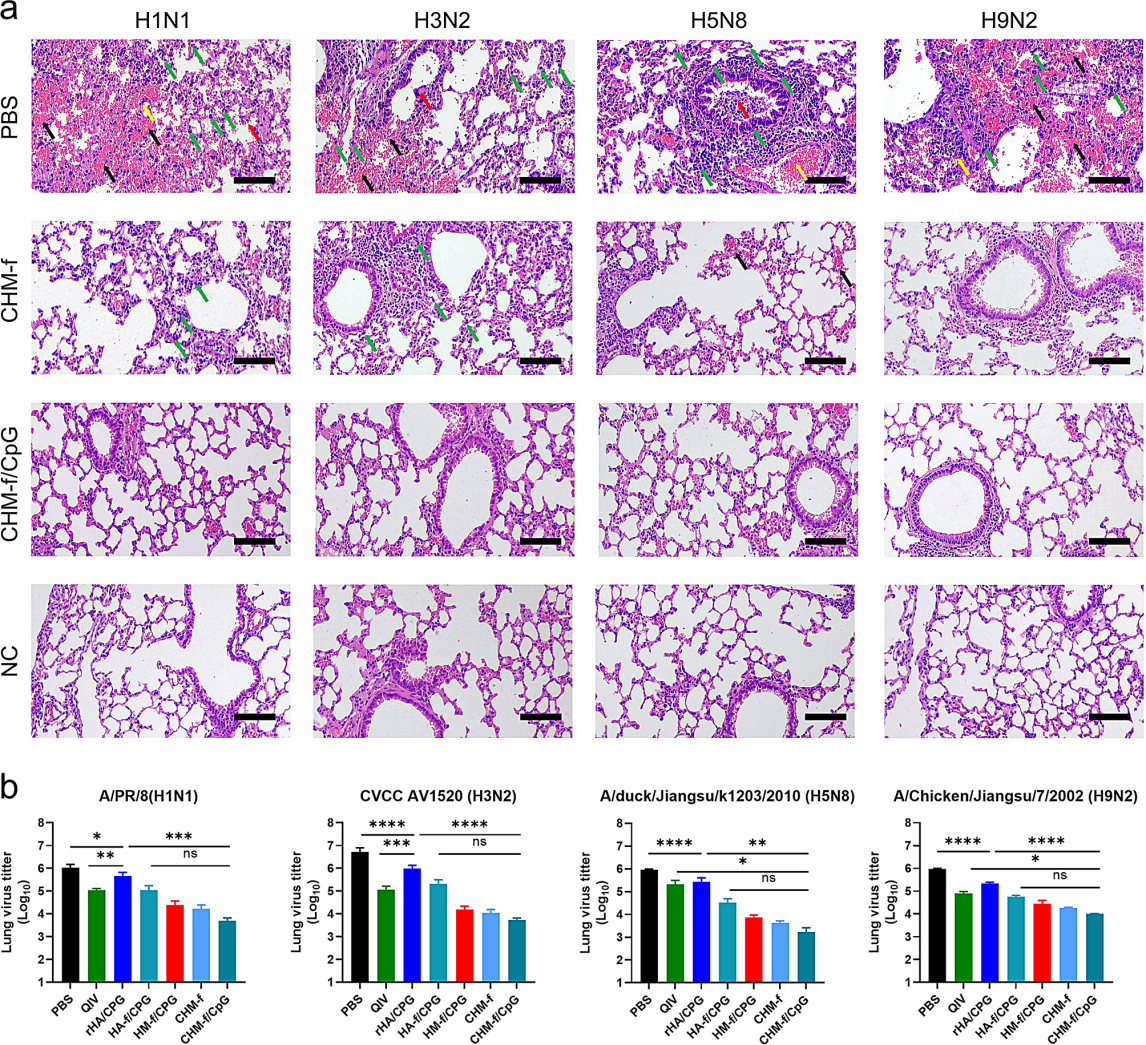
Lung physiology on Day 5 postinfection. (**a**) Histological analysis. The black arrows indicate hemorrhage. Red arrows indicate bronchial epithelial cell detachment and necrosis. Yellow arrows indicate alveolar wall thickening. The green arrows indicate inflammatory cell infiltration. The gold arrows indicate congestion (scale bars, 100 μm). (**b**) Determination of lung virus titres. The data are expressed as the means ± SEMs (*n* = 4, *P < 0.05, **P < 0.01, ***P < 0.001, ****P < 0.0001, ns, not significant)

To assess lung infection, lung tissues were collected from the mice 5 days post challenge, and we performed histological analysis as well as lung viral titre assays. As shown in Fig. 9a and S6, the lung tissues of the mice immunized with PBS, QIV, rHA/CpG or HA-f/CpG presented bronchial epithelial cell detachment and necrosis, hemorrhage, congestion, alveolar wall thickening, and inflammatory cell infiltration. The lungs of the mice in the HM-f/CpG- or CHM-f-immunized groups experienced slight pathological changes. In contrast, no obvious pathological changes were observed in the mice immunized with CHM-f/CpG (Table S1). Additionally, the lung virus titres of the mice in all immunized groups were significantly lower than those of the PBS control group. Importantly, CHM-f/CpG induced the lowest lung virus titres (Fig. 9b). These results demonstrated that CHM-f nanoparticle immunization protected mouse lungs from viral attack, reduced the lung viral load and maintained lung tissue integrity.

## Discussion

Due to the continuous antigenic drift and shift of influenza viruses, universal influenza vaccines with broad-spectrum protection are urgently needed. The respiratory mucosa is the first line of defense against pathogens, and an effective mucosal immune response provides cross-immune protection against homologous and heterologous influenza viruses. Intranasal immunization is a safe and effective way to induce mucosal immunity and resist influenza virus infection. Studies have shown that nasal immunization confers cross-protection against divergent virus infections by inducing highly effective local mucosal immune responses in the respiratory tract and robust humoral and cellular immune responses [8, 46]. However, the natural barrier of the respiratory mucosa and mucociliary clearance make the transepithelial transport of nasal vaccines extremely difficult. The nanoparticle system promotes antigen transport across the mucosal barrier, protects antigens from protease degradation, and enhances the adjuvant activity of vaccines to nasal lymphoid tissues. It, therefore, can be used as an antigen delivery platform for intranasal vaccines [47–49]. Previous reports have shown that nanoparticle vaccines can efficiently accumulate in lymph nodes to enhance immune processing [50–52]. And nanoparticle vaccines are easily captured by dendritic cells (DCs) and macrophages, which process and present antigens to the CD4 T helper cells, followed by T follicular helper (Tfh) cells and B cells coordination to enhance the antibody responses [53, 54]. In our research, we found that CHM-f nanoparticles efficiently induced DCs maturation and upregulated the expression of the surface molecules on DCs, which is necessary for subsequent T-cell activation. And the CHM-f nanoparticles can be effectively phagocytosed by DCs, enhancing antigen presentation in the lymph nodes, which is consistent with previous findings that the efficiency of DCs uptake of nanoparticles has significantly increased compared with soluble antigens alone [15].

Ferritin is an iron storage protein widely found in living organisms. Ferritin itself has some immunological activity and can enhance the immune response by activating immune cells and promoting antibody production. In addition, the presentation of antigens on the surface of ferritin has many desirable properties, such as the ability of ferritin, which consists of 24 subunits, to present antigens uniformly at high density, and the remarkable thermal and pH stability of ferritin nanocages [26]. Due to their size (10 ~ 12 nm), ferritin nanocages can be readily taken up by macrophages and dendritic cells (DCs) for migration to the lymph node to enhance cellular and humoral immune responses [55]. Given these advantages, ferritin-based vaccines have been demonstrated to be remarkably potent and can be utilized not only for infectious diseases but also for cancer and autoimmune disease vaccines [17–22]. In this study, we developed a broadly protective influenza nanoparticle vaccine for intranasal immunization, CHM-f. CHM-f nanoparticles incorporate the ectodomain of the HA protein, three repeated M2e epitopes and the M-cell-targeting ligand Co4B, and utilize a ferritin nanoparticle platform for antigen delivery. The results of intranasal immunization revealed that CHM-f induced not only strong systemic cellular and humoral immunity but also local mucosal immune responses and provided cross-protection against lethal challenges caused by multiple influenza strains.

Critical targets in our design of universal influenza vaccines included the most important protective antigens of influenza virus, HA proteins, which induce neutralizing antibodies, and the highly conserved M2e peptides, which induce cross-protection. Enhancing the strength and breadth of antibody responses helps combat potentially immune-escape influenza strains. We found that immunization with CHM-f nanoparticle groups (CHM-f/CpG and CHM-f) significantly increased systemic and local mucosal antigen-specific antibody responses. Compared with the HA-f and HM-f nanoparticle groups, the CHM-f nanoparticle groups presented significantly increased antigen-specific IgG levels, HAI titres, and neutralizing antibody titres in the serum, especially compared with the rHA and QIV groups. We also found notably elevated cross-neutralizing antibody titres against different IAVs, including H1N1, H3N2, H5N8, and H9N2 viruses, indicating cross-protection. The high level of cross-neutralizing antibodies induced by the CHM-f nanoparticle groups may have benefitted from the following factors. Nanoparticles rapidly accumulate in lymph nodes and stay longer for better antigen delivery to APCs [56, 57]. The surface of nanoparticles has a relatively high density of antigens that mimic natural viruses and react with pattern recognition receptors (PRRs) on the surface of immune cells. Also, Co4B peptides, which are M-cell-targeting ligands, enhance antigen delivery to the nasal mucosa and enhance systemic and mucosal local immune responses [29, 30]. These factors suggest that ferritin nanoparticles can serve as a self-adjuvant vaccine platform, and the M-cell-targeting ligand-conjugated Ag can significantly enhance mucosal and systemic immune responses.

The most important antibody involved in mucosal immunity, IgA, plays a critical role in the defense against influenza infection. As mucosal sIgA may produce more broadly reactive antibodies than IgG [58], the sIgA induced by the CHM-f nanoparticle groups also plays a key role in protection against multiple subtypes of influenza virus infection. We observed that CHM-f nanoparticle vaccination potently increased epitope-specific sIgA titres in mucosal washes, indicating that the breadth of antibody responses was improved.

In addition to humoral immunity, T-cell-mediated cellular immunity plays crucial roles in resistance to influenza virus infection [59]. In our study, we found that the CHM-f nanoparticles induced strong cellular immune responses and significantly increased the percentages of CD4^+^ T and CD8^+^ T cells. CD4^+^ T cells can promote B-cell activation, differentiation, and antibody production mainly through the secretion of effector cytokines and contact with B cells; they can also promote the activation of Tc cells and enhance the ability of Tc cells to kill target cells. CD8^+^ T cells are mainly cytotoxic T cells (TCs), and during the immune effector phase, activated T cells produce CTLs through contact-dependent lysis of virus-invaded cells [60–62]. T helper cells are an indispensable subset of the immune response in vivo, and their main function is to secrete effector cytokines to assist other immune cells in their function. We found that CHM-f nanoparticles significantly increased the number of IL-2-, IL-4-, and CD40L-secreting cells in mouse spleen lymph nodes. IL-2, IL-4 and CD40L are important factors that promote the proliferation and differentiation of B lymphocytes into antibody-secreting plasma cells. The main role of IFN-γ is to modulate cellular immunity and to inhibit viral replication. We also observed significantly increased IFN-γ secretion, which contributed to protection against various influenza virus strains during CHM-f nanoparticle vaccination.

Another interesting and inspiring point is that the CHM-f nanoparticles provided complete protection against four divergent virus subtypes of IAV, including H1N1, H3N2, H5N8 and H9N2. They are the primary contributors to influenza virus diversity. Subtypes H1N1 and H3N2 of IAV are the dominant strains in seasonal influenza epidemics and pose a significant burden on human healthcare. Subtypes H5N8 and H9N2 usually occur in poultry and wild birds, and once cross-species transmission occurs, they can pose a pandemic risk and a significant threat to human health [63–65]. The CHM-f nanoparticle vaccine demonstrated the feasibility of eliciting broad heterosubtypic IAV protection and can be a promising candidate as a universal influenza vaccine.

The efficacy of existing seasonal influenza vaccines is not particularly satisfactory. Adjuvant use was considered as an approach to improve influenza vaccine performance. MF59 is the only adjuvant approved for seasonal influenza vaccine. CpG is considered an effective adjuvant for its ability to enhance the mucosal immune response and increase the speed and magnitude of vaccine response [66–68], as verified by our previous studies [35, 69]. In 2017, CpG 1018 was approved for use in hepatitis B vaccines in adults 18 years of age and older [70]. CpG 1018-adjuvanted COVID-19 vaccine was authorized for emergency use in adults 18 years of age and older [71]. The CpG used in this study belongs to the B-type, a potent Th1 adjuvant. It induces plasmacytoid dendritic cell (pDC) differentiation and production of tumor necrosis factor α (TNF-α), and strongly stimulates B cell to proliferate and secrete immunoglobulins or cytokines [72]. In the present study, the addition of CpG IAMA-002 significantly improved the immunization effect of the vaccine, and the CHM-f/CpG nanoparticle-immunized mice exhibited less weight loss and were fully protected against lethal challenge by four divergent subtypes of influenza viruses. The important finding of this study is that we demonstrated ferritin nanoparticle influenza vaccines CHM-f, formulated with or without CpG adjuvant, provided cross-protection against divergent strains of influenza virus. The various formulations should help to meet the regulatory requirements for protecting different target populations against influenza infection.

## Conclusion

In conclusion, we developed an intranasal HA chimeric multiepitope CHM-f nanoparticle vaccine. Mice intranasally immunized with the nanoparticle vaccine with or without CpG adjuvant induced robust, broadly protective immune responses, conferring complete protection against homologous and heterologous influenza strains. This vaccine can be rapidly mass produced in a baculovirus expression vector system (BEVS) in response to an influenza pandemic. BEVS is considered one of the most efficient platforms for producing recombinant proteins, but there are still issues with protein stability and quality, and insufficient glycosylation remains the biggest issue with this system. Baculovirus genome modifications are being performed to improve protein expression and quality. Additionally, insect cell lines are being modified to enhance protein folding and glycosylation. The use of enhanced optimization of cell culture, improved culture media, and exploration of production processes are improving the capabilities of BEVS. Through the above optimization, it will make the BEVS system further mature, providing unmet needs in producing biologically active proteins. This study also provides a promising candidate for a universal influenza vaccine. Immune efficacy in large animal models needs to be further evaluated.

## Electronic supplementary material

Below is the link to the electronic supplementary material.

Supplementary Material 1: Additional file 1: figure S1 Stability evaluation of the influenza nanoparticle vaccines. (a) SDS-PAGE analysis of the stability of CHM-f nanoparticle vaccine stored at 4 °C and − 80 °C for 180 days. (b) Determination of HA titres, protein concentration and purity of CHM-f nanoparticles stored at 4 °C for 45 days. (c) Determination of HA titres, protein concentration and purity of CHM-f nanoparticles stored at -80 °C for 45 days.

Supplementary Material 2: Additional file 2: figure S2 Safety evaluation post-vaccination with CHM-f nanoparticles. (a) Body weight changes for 7 days post-vaccination. (b) Lung histological analysis for 7 days post-vaccination.

Supplementary Material 3: Additional file 3: figure S3 Detection of mucosal IgA antibodies in mice two months after booster immunization. (a) Anti-HA IgA levels in nasal wash samples. (b) Anti-M2e IgA levels in nasal wash samples. (c) Anti-HA IgA levels in lung washes. (d) Anti-M2e IgA levels in lung washes.

Supplementary Material 4: Additional file 4: figure S4 Gating strategy for flow cytometry analysis of T lymphocytes in the spleen.

Supplementary Material 5: Additional file 5: figure S5 Representative flow cytometry analysis results of the percentages of CD3^+^CD4^+^ and CD3^+^CD8^+^ T cells.

Supplementary Material 6: Additional file 6: figure S6 Histological pathology analysis of the lungs of mice immunized with QIV, rHA/CpG, HA-f/CpG, or HM-f/CpG on Day 5 postinfection.

Supplementary Material 7: Additional file 7: table S1 Histopathological scoring of lungs on Day 5 postinfection. (a) Definition of the grading of five scoring systems. (b) Histopathological scoring of lungs.

Supplementary Material 8: Additional file 8: table S2 Evaluation of the HA- and M2e-specific IgG endpoint titres in mice.

## Data Availability

No datasets were generated or analysed during the current study.
