# Supplementary figures and images for: Cross-protection against homo and heterologous influenza viruses via intranasal administration of an HA chimeric multiepitope nanoparticle vaccine
Source: J Nanobiotechnology. 2025 Feb 4;23:77. doi: 10.1186/s12951-025-03122-6 (PMC11792681; doi:10.1186/s12951-025-03122-6)

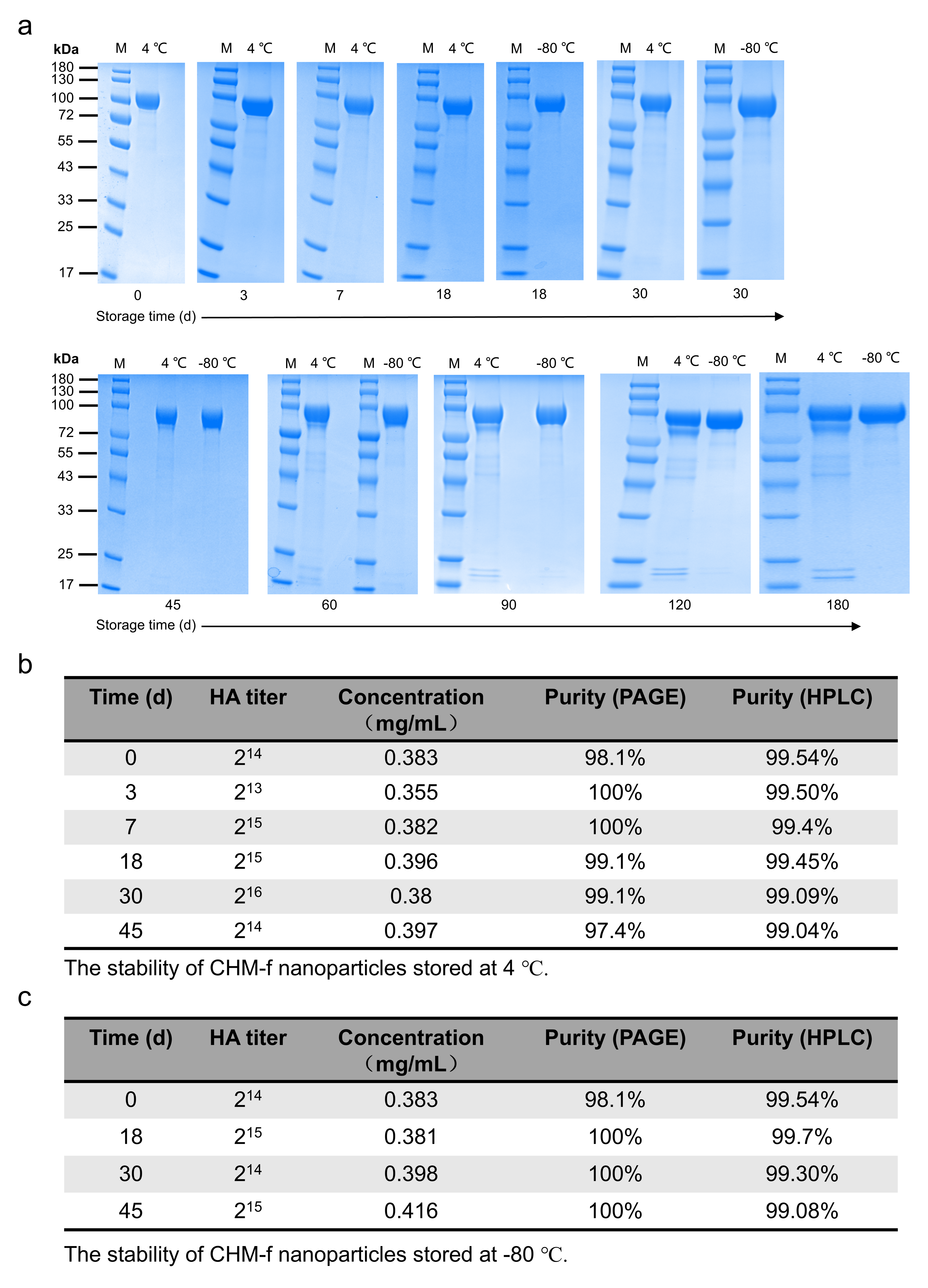

Supplement: Supplementary file 1 — Supplementary Material 1: Additional file 1: figure S1 Stability evaluation of the influenza nanoparticle vaccines. (a) SDS-PAGE analysis of the stability of CHM-f nanoparticle vaccine stored at 4 °C and − 80 °C for 180 days. (b) Determination of HA titres, protein concentration and purity of CHM-f nanoparticles stored at 4 °C for 45 days. (c) Determination of HA titres, protein concentration and purity of CHM-f nanoparticles stored at -80 °C for 45 days. [file 12951_2025_3122_MOESM1_ESM.tif]

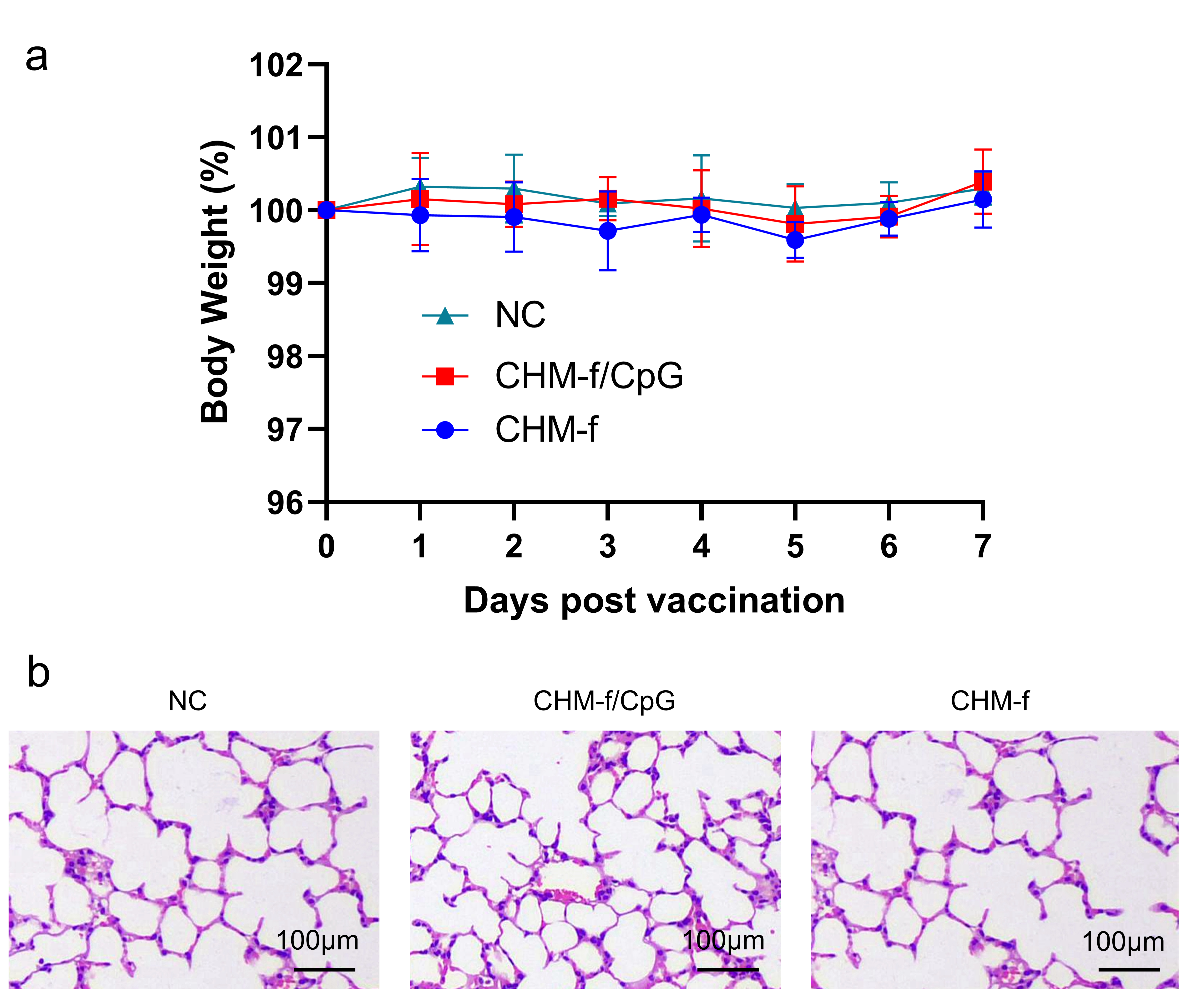

Supplement: Supplementary file 2 — Supplementary Material 2: Additional file 2: figure S2 Safety evaluation post-vaccination with CHM-f nanoparticles. (a) Body weight changes for 7 days post-vaccination. (b) Lung histological analysis for 7 days post-vaccination. [file 12951_2025_3122_MOESM2_ESM.tif]

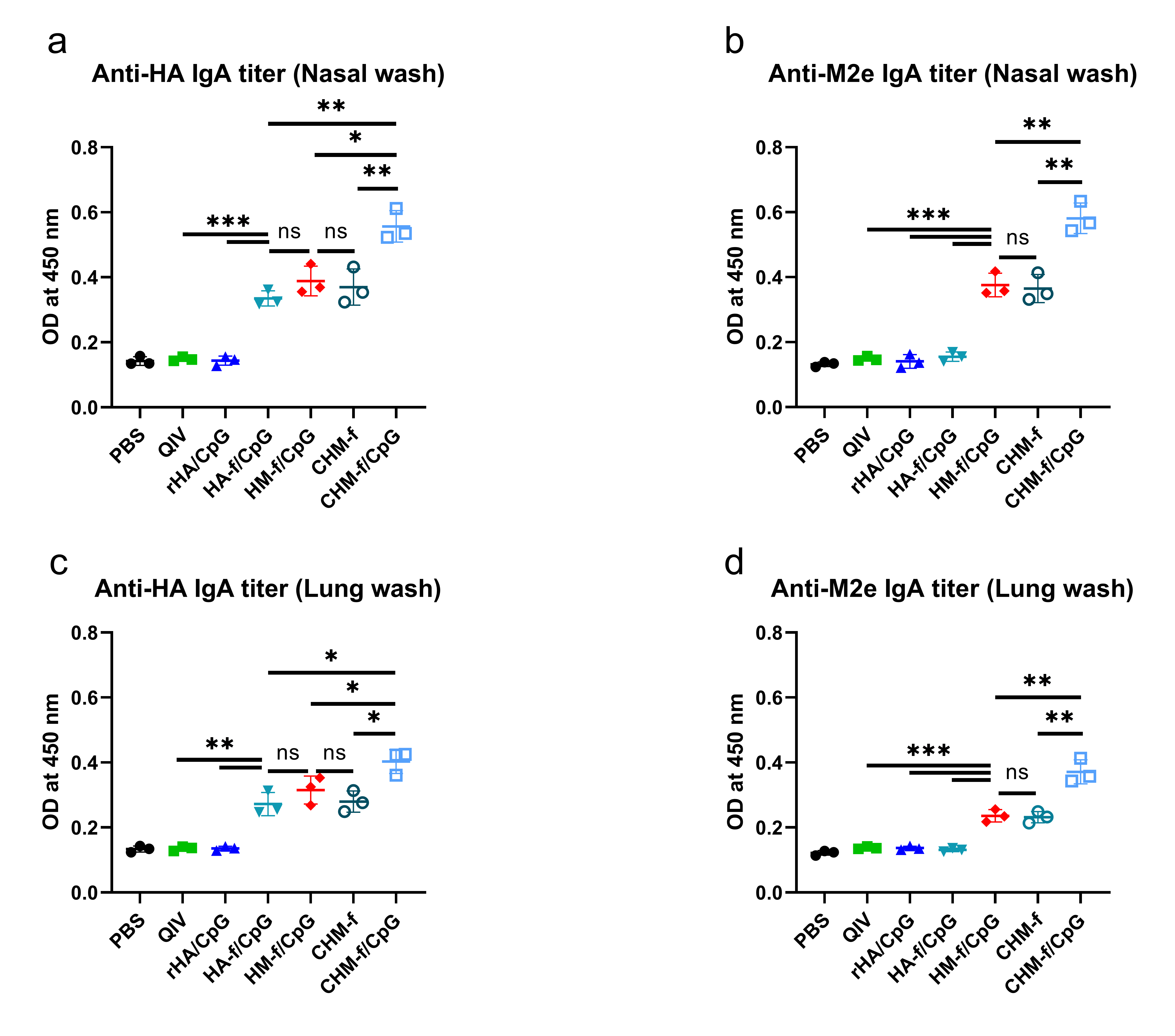

Supplement: Supplementary file 3 — Supplementary Material 3: Additional file 3: figure S3 Detection of mucosal IgA antibodies in mice two months after booster immunization. (a) Anti-HA IgA levels in nasal wash samples. (b) Anti-M2e IgA levels in nasal wash samples. (c) Anti-HA IgA levels in lung washes. (d) Anti-M2e IgA levels in lung washes. [file 12951_2025_3122_MOESM3_ESM.tif]

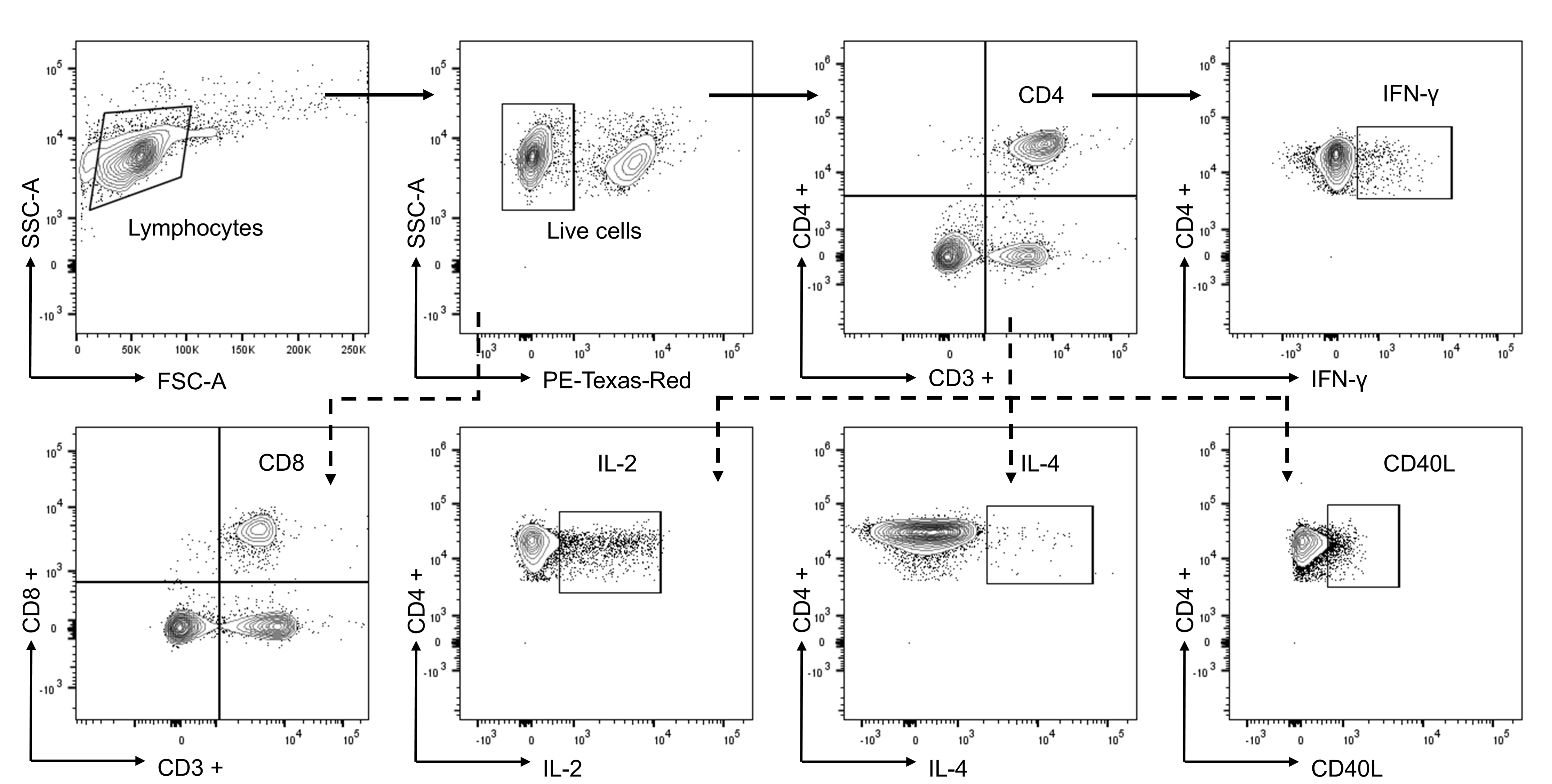

Supplement: Supplementary file 4 — Supplementary Material 4: Additional file 4: figure S4 Gating strategy for flow cytometry analysis of T lymphocytes in the spleen. [file 12951_2025_3122_MOESM4_ESM.tif]

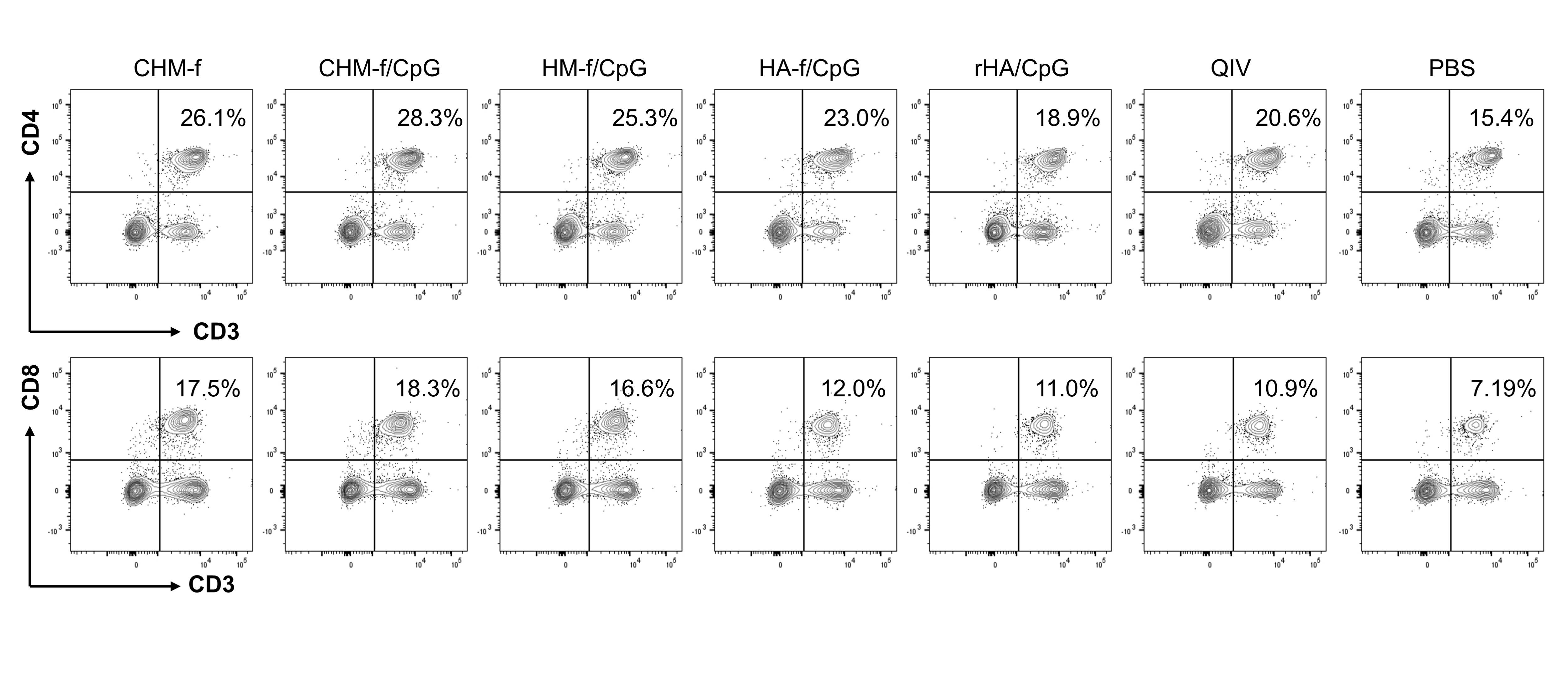

Supplement: Supplementary file 5 — Supplementary Material 5: Additional file 5: figure S5 Representative flow cytometry analysis results of the percentages of CD3+CD4+ and CD3+CD8+ T cells. [file 12951_2025_3122_MOESM5_ESM.tif]

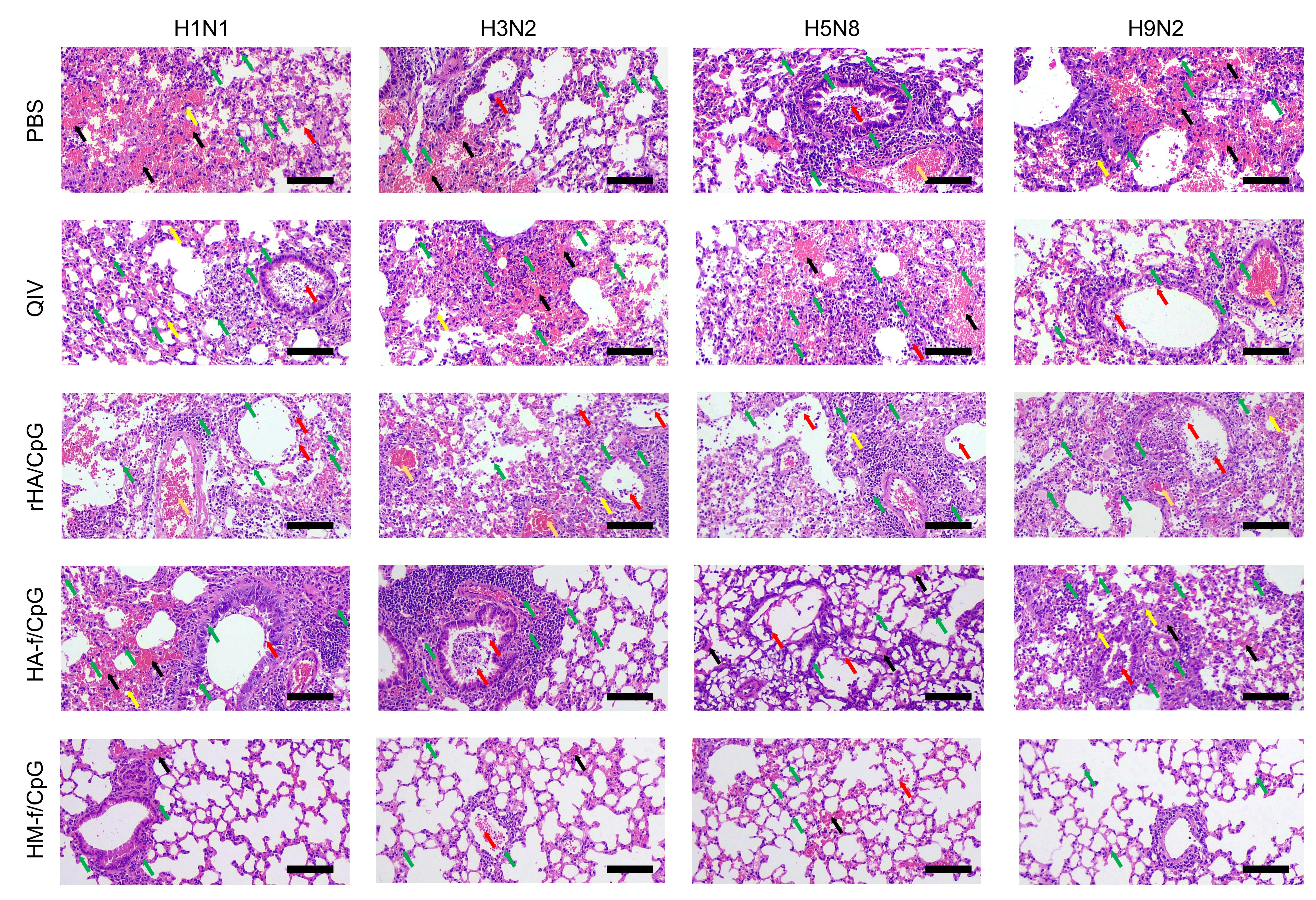

Supplement: Supplementary file 6 — Supplementary Material 6: Additional file 6: figure S6 Histological pathology analysis of the lungs of mice immunized with QIV, rHA/CpG, HA-f/CpG, or HM-f/CpG on Day 5 postinfection. [file 12951_2025_3122_MOESM6_ESM.jpg]
