# Supplementary material for: Cross-protection against homo and heterologous influenza viruses via intranasal administration of an HA chimeric multiepitope nanoparticle vaccine
Source: J Nanobiotechnology. 2025 Feb 4;23:77. doi: 10.1186/s12951-025-03122-6 (PMC11792681; doi:10.1186/s12951-025-03122-6)
Supplement: Supplementary file 7 — Supplementary Material 7: Additional file 7: table S1 Histopathological scoring of lungs on Day 5 postinfection. (a) Definition of the grading of five scoring systems. (b) Histopathological scoring of lungs. [file 12951_2025_3122_MOESM7_ESM.docx]

**Table S1a.** Definition of the grading of five scoring systems.

| Scoring | Lesion level | Definition of grading |
| --- | --- | --- |
| 0（-） | normal | Under experimental conditions, considered in conjunction with factors such as age, sex, and germline, alterations may have occurred that might otherwise be considered as deviations from the normal state |
| 1（+） | slight | Changes occurring that barely exceed those within the normal range (i.e., minimal changes) |
| 2（++） | mild | Lesions are easily identifiable but limited in severity; lesions may not produce any dysfunction; lesions range from 11% to 20% of the tissue examined |
| 3（+++） | moderate | Lesions are prominent, with a likely tendency to progress toward severity. May produce limited tissue or organ dysfunction; 21% to 40% of tissues involved |
| 4（++++） | severe | Severe and complete lesions that are expected to produce significant tissue or organ dysfunction; lesions involving 41% to 100% of the examined tissue area |

**Table S1b.** Histopathological scoring of lungs on Day 5 postinfection.

| Groups | Challenge Strains | Results | | | | |
| --- | --- | --- | --- | --- | --- | --- |
|  |  | bronchial epithelial cell detachment and necrosis | alveolar wall thickening | inflammatory cell infiltration | hemorrhage | congestion |
| NC | / | normal | normal | normal | normal | + |
| PBS | H1N1 | +++ | +++ | +++ | ++++ | ++ |
|  | H3N2 | +++ | ++++ | +++ | +++ | +++ |
|  | H5N8 | ++++ | ++++ | ++++ | ++ | ++ |
|  | H9N2 | ++++ | ++++ | ++++ | ++ | ++ |
| QIV | H1N1 | +++ | +++ | +++ | ++ | + |
|  | H3N2 | ++ | +++ | +++ | +++ | ++ |
|  | H5N8 | +++ | +++ | +++ | +++ | ++ |
|  | H9N2 | +++ | ++ | +++ | ++ | +++ |
| rHA/CpG | H1N1 | +++ | +++ | +++ | ++ | +++ |
|  | H3N2 | +++ | ++++ | +++ | ++ | +++ |
|  | H5N8 | +++ | +++ | +++ | ++ | ++ |
|  | H9N2 | ++++ | +++ | +++ | ++ | +++ |
| HA-f/CpG | H1N1 | ++ | +++ | +++ | ++ | ++ |
|  | H3N2 | +++ | ++ | +++ | + | ++ |
|  | H5N8 | ++ | ++ | ++ | ++ | ++ |
|  | H9N2 | +++ | +++ | +++ | ++ | ++ |
| HM-f/CpG | H1N1 | + | ++ | + | + | ++ |
|  | H3N2 | ++ | ++ | ++ | + | ++ |
|  | H5N8 | ++ | ++ | ++ | ++ | ++ |
|  | H9N2 | + | ++ | ++ | + | + |
| CHM-f | H1N1 | ++ | ++ | ++ | + | ++ |
|  | H3N2 | + | ++ | ++ | + | ++ |
|  | H5N8 | ++ | ++ | ++ | ++ | + |
|  | H9N2 | ++ | ++ | ++ | + | + |
| CHM-f/CpG | H1N1 | normal | + | normal | + | + |
|  | H3N2 | normal | + | normal | normal | ++ |
|  | H5N8 | normal | + | + | + | + |
|  | H9N2 | + | + | + | normal | + |
