# Supplementary material for: Cross-protection against homo and heterologous influenza viruses via intranasal administration of an HA chimeric multiepitope nanoparticle vaccine
Source: J Nanobiotechnology. 2025 Feb 4;23:77. doi: 10.1186/s12951-025-03122-6 (PMC11792681; doi:10.1186/s12951-025-03122-6)
Supplement: Supplementary file 8 — Supplementary Material 8: Additional file 8: table S2 Evaluation of the HA- and M2e-specific IgG endpoint titres in mice. [file 12951_2025_3122_MOESM8_ESM.docx]

Table S2a Evaluation of the HA-specific IgG endpoint titres in mice.

| Days  Groups | 21 days (Prime) | | | | | | 49 days (Boost) | | | | | |
| --- | --- | --- | --- | --- | --- | --- | --- | --- | --- | --- | --- | --- |
| PBS | 10 | 10 | 10 | 10 | 10 | 10 | 10 | 10 | 10 | 10 | 10 | 10 |
| QIV | 1000 | 1000 | 1000 | 2000 | 2000 | 1000 | 4000 | 4000 | 4000 | 4000 | 2000 | 2000 |
| rHA | 200 | 400 | 200 | 400 | 400 | 400 | 800 | 800 | 400 | 800 | 400 | 800 |
| HA-f | 1600 | 800 | 1600 | 3200 | 1600 | 1600 | 6400 | 1600 | 6400 | 6400 | 3200 | 6400 |
| HM-f | 6400 | 1600 | 3200 | 3200 | 1600 | 6400 | 12800 | 3200 | 6400 | 6400 | 6400 | 12800 |
| CHM-f | 51200 | 51200 | 25600 | 25600 | 51200 | 51200 | 102400 | 51200 | 51200 | 51200 | 102400 | 102400 |
| rHA/CpG | 1000 | 2000 | 1000 | 2000 | 2000 | 2000 | 4000 | 8000 | 2000 | 4000 | 4000 | 4000 |
| HA-f/CpG | 32000 | 16000 | 8000 | 32000 | 16000 | 16000 | 64000 | 32000 | 64000 | 64000 | 32000 | 32000 |
| HM-f/CpG | 16000 | 16000 | 32000 | 8000 | 32000 | 8000 | 32000 | 32000 | 128000 | 32000 | 64000 | 16000 |
| CHM-f/CpG | 32000 | 64000 | 32000 | 32000 | 32000 | 64000 | 64000 | 128000 | 128000 | 64000 | 128000 | 128000 |

Table S2b Evaluation of the M2e-specific IgG endpoint titres in mice.

| Days  Groups | 21 days (Prime) | | | | | | 49 days (Boost) | | | | | |
| --- | --- | --- | --- | --- | --- | --- | --- | --- | --- | --- | --- | --- |
| PBS | 10 | 10 | 10 | 10 | 10 | 10 | 10 | 10 | 10 | 10 | 10 | 10 |
| QIV | 10 | 10 | 10 | 10 | 10 | 10 | 10 | 10 | 10 | 10 | 10 | 10 |
| rHA | 10 | 10 | 10 | 10 | 10 | 10 | 10 | 10 | 10 | 10 | 10 | 10 |
| HA-f | 10 | 10 | 10 | 10 | 10 | 10 | 10 | 10 | 10 | 10 | 10 | 10 |
| HM-f | 3200 | 1600 | 1600 | 1600 | 800 | 3200 | 6400 | 3200 | 1600 | 1600 | 1600 | 6400 |
| CHM-f | 25600 | 25600 | 12800 | 12800 | 51200 | 51200 | 102400 | 51200 | 25600 | 51200 | 51200 | 102400 |
| rHA/CpG | 10 | 10 | 10 | 10 | 10 | 10 | 10 | 10 | 10 | 10 | 10 | 10 |
| HA-f/CpG | 10 | 10 | 10 | 10 | 10 | 10 | 10 | 10 | 10 | 10 | 10 | 10 |
| HM-f/CpG | 16000 | 8000 | 32000 | 8000 | 16000 | 16000 | 32000 | 16000 | 32000 | 32000 | 64000 | 32000 |
| CHM-f/CpG | 32000 | 32000 | 32000 | 16000 | 64000 | 32000 | 128000 | 32000 | 64000 | 64000 | 128000 | 128000 |
